# AupA and AupB Are Outer and Inner Membrane Proteins Involved in Alkane Uptake in Marinobacter hydrocarbonoclasticus SP17

**DOI:** 10.1128/mBio.00520-18

**Published:** 2018-06-05

**Authors:** Julie Mounier, Florence Hakil, Priscilla Branchu, Muriel Naïtali, Philippe Goulas, Pierre Sivadon, Régis Grimaud

**Affiliations:** a CNRS/Univ Pau et Pays Adour, Institut des Sciences Analytiques et de Physico-Chimie pour l’Environnement et les Matériaux, UMR5254, Pau, France; b MICALIS, UMR1319, Bioadhésion Biofilms et Hygiène des Matériaux, INRA, AgroParisTech, Massy, France; University of Georgia

**Keywords:** alkanes, biodegradation, biofilms, marine microbiology

## Abstract

This study describes the functional characterization of two proteins, AupA and AupB, which are required for growth on alkanes in the marine hydrocarbonoclastic bacterium Marinobacter hydrocarbonoclasticus. The *aupA* and *aupB* genes form an operon whose expression was increased upon adhesion to and biofilm formation on *n-*hexadecane. AupA and AupB are outer and inner membrane proteins, respectively, which are able to interact physically. Mutations in *aupA* or/and *aupB* reduced growth on solid paraffin and liquid *n-*hexadecane, while growth on nonalkane substrates was not affected. In contrast, growth of *aup* mutants on *n-*hexadecane solubilized in Brij 58 micelles was completely abolished. Mutant cells had also lost the ability to bind to *n*-hexadecane solubilized in Brij 58 micelles. These results support the involvement of AupA and AupB in the uptake of micelle-solubilized alkanes and provide the first evidence for a cellular process involved in the micellar uptake pathway. The phylogenetic distribution of the *aupAB* operon revealed that it is widespread in marine hydrocarbonoclastic bacteria of the orders *Oceanospirillales* and *Alteromonadales* and that it is present in high copy number (up to six) in some *Alcanivorax* strains. These features suggest that Aup proteins probably confer a selective advantage in alkane-contaminated seawater.

## IMPORTANCE

Bacteria are the main actors of the biological removal of hydrocarbons in seawater, and so, it is important to understand how they degrade hydrocarbons and thereby mitigate marine environmental damage. Despite a considerable amount of literature about the dynamic of microbial communities subjected to hydrocarbon exposure and the isolation of strains that degrade hydrocarbons, most of the genetic determinants and molecular mechanisms of bacterial hydrocarbon uptake remain unknown. This study identifies two genes, *aupA* and *aupB*, in the hydrocarbonoclastic bacterium Marinobacter hydrocarbonoclasticus that are present frequently in multiple copies in most of the marine hydrocarbon-degrading bacteria for which the genomic sequence is available. AupA and AupB are two novel membrane proteins interacting together that are involved in the uptake of alkanes dissolved in surfactant micelles. The function and the phylogenetic distribution of *aupA* and *aupB* suggest that they might be one attribute of the remarkable adaptation of marine hydrocarbonoclastic bacteria that allow them to take advantage of hydrocarbons.

## INTRODUCTION

The great energetic value of hydrocarbons, their high carbon content, and their omnipresence in the marine environment make them valuable substrates for marine heterotrophic bacteria. It is therefore not surprising that a large number of hydrocarbon-degrading strains have been isolated from various marine environments and that hydrocarbon degradation genes have been found in the genomes of many marine strains, even in those not isolated for their hydrocarbon degradation capabilities (1, 2). Using culture-independent approaches, it was observed that some bacteria became dominant in marine environments contaminated by an oil spill or in micro- or mesocosms that mimic these environments while they were barely detectable in pristine environments (1, 2). These bacteria belong to only a few genera such as *Alcanivorax*, *Marinobacter*, *Cycloclasticus*, *Oleiphilus*, *Oleispira*, and *Thalassolituus*. This group of bacteria, referred to as the marine hydrocarbonoclastic bacteria (HCB), has been recognized as comprising the most important hydrocarbon degraders in the marine environment. Marine HCB are so-called specialists because they exhibit a strong bias toward hydrocarbons in their substrate range (3).

Because of their strong hydrophobic character, hydrocarbons are poorly soluble in the aqueous phase and tend to partition into organic phases or to adsorb to particles. Therefore, the assimilation of hydrocarbons entails coping with their poor bioavailability (4, 5). Biochemical and physiological studies revealed that bacteria have indeed evolved physiological processes to access hydrocarbons, such as biosurfactant synthesis and biofilm formation (6, 7). Various strains of *Alcanivorax* sp. produce glycolipid or proline-lipid biosurfactants during growth on hydrocarbons, and in some cases, these molecules have been shown to promote growth on alkanes (8, 9). The production of surface-active compounds was also observed in *Acinetobacter* species. Acinetobacter calcoaceticus RAG-1 produces emulsan, a lipopolysaccharide essential for the growth on crude oil, while Acinetobacter radioresistens KA53 excretes alasan, a polysaccharide-protein complex, which solubilizes polycyclic aromatic hydrocarbons (10, 11). In M. hydrocarbonoclasticus SP17, surface-active molecules are released simultaneously with the binding of the cells to the *n-*hexadecane–water interface, resulting in the formation of an interfacial viscoelastic film (12). Although these compounds have not been identified, their behavior at the interface suggests that they are high-molecular-weight molecules forming a network at the interface reminiscent of an extracellular matrix (12, 13). Biofilm formation at hydrocarbon-water interfaces is another adaptive process that allows bacteria to assimilate nearly water-insoluble substrates. Such biofilms have been observed with many strains like M. hydrocarbonoclasticus SP17, Alcanivorax borkumensis SK2, and Oleiphilus messinensis ME102 or consortia degrading hydrocarbons, and in a few examples, they have been shown to increase the rate of mass transfer of hydrocarbons from the organic phase to the cell (14–18).

Surfactant production and biofilm formation are major processes allowing access to alkanes by enhancing the mass transfer of the hydrocarbons from the organic phase to the cell surface. However, once they have reached the cell vicinity, hydrocarbon molecules must be transported into the cell’s interior. In the case of alkanes, this uptake process remains poorly understood. The outer membrane (OM) of Gram-negative bacteria forms a barrier that hinders the passage of both hydrophilic and hydrophobic compounds (19). Consequently, transport of alkanes must therefore be performed by proteins that facilitate diffusion across the OM. To date, AlkL of Pseudomonas putida GPo1 is the only protein for which clear evidence for a role in alkane transport has been obtained (20, 21). AlkL exhibits sequence similarity to OmpW that is suspected to mediate the uptake of small hydrophobic molecules by a lateral diffusion mechanism similar to that of the Escherichia coli long-chain fatty acid transporter FadL (22, 23). FadL is an outer membrane β-barrel protein that binds long-chain fatty acids with a weak affinity. After binding, the substrate diffuses into the OM through a lateral opening in the wall of the barrel. The FadL protein alone is sufficient to carry out this transport and does not require any external energy source (23).

In cultures of M. hydrocarbonoclasticus SP17 with hydrophobic organic compounds (HOCs) as the sole carbon and energy sources, growth occurs exclusively as biofilms at the HOC-water interfaces, whereas biofilms are hardly detected on nonmetabolizable alkanes such as heptamethylnonane (HMN) or inert substrata like plastic or glass in the presence of a soluble carbon source like acetate or lactate. Furthermore, the kinetics of biofilm formation and *n-*hexadecane degradation established that these two processes are interrelated (13). To obtain a more comprehensive picture of biofilm development at the hydrocarbon-water interface in the marine environment, proteomic studies were conducted on the biofilm of M. hydrocarbonoclasticus SP17 growing on *n-*hexadecane (24, 25). It revealed that biofilm cells expressed a specific proteome in which 50% of the detected proteins had their quantity levels altered compared to planktonic cells growing exponentially on acetate. This indicates that cells undergo a profound reshaping of their physiology when forming a biofilm at the *n-*hexadecane–water interface. This study pinpointed two proteins, encoded by the *MARHY0478* and *MARHY0477* coding sequences (CDS), which were strongly overproduced under biofilm conditions compared to cells growing on acetate. The MARHY0478 protein possesses a conserved domain belonging to the family of the outer membrane transporters of hydrophobic compounds of which FadL is the archetype. The protein MARHY0477 was annotated as a conserved protein of unknown function (24). Here, we present evidence indicating that these two proteins are involved together in alkane uptake. MARHY0478 and MARHY0477 were consequently renamed AupA and AupB (alkane uptake protein), respectively.

## RESULTS

### *aupA* and *aupB* form an operon whose expression is increased in the presence of alkanes.

The two *aupA* and *aupB* genes are adjacent on the chromosome of M. hydrocarbonoclasticus SP17 and orientated in the same direction of transcription, being separated by only 11 nucleotides. Moreover, a sigma-70 promoter located upstream of *aupA* and a rho-independent transcription terminator located downstream of *aupB* have been predicted (see Fig. S1A in the supplemental material). Together, these features indicate that *aupA* and *aupB* could constitute an operon. The cotranscription of these two genes was demonstrated by PCR amplification of a DNA fragment overlapping *aupA* and *aupB* CDS using cDNAs as the template (Fig. S1B).

10.1128/mBio.00520-18.2 FIG S1  Genomic organization of the *aup* locus. (A) Genetic structure of the *aupAB* operon. Protein sequences are in gray, and nucleotide sequences are in black. (B) Agarose gel electrophoresis of RT-PCR amplifications of the *aupAB* region. The positions on the genome of the primers used are indicated. PCR products were obtained with primers 0478F-Bam and 0478deltaR (electrophoresis 1), 0478F and 0477R (electrophoresis 2), and 0477-F and Op0476R (electrophoresis 3). DNA templates used were as follows: lane (+), 10 ng of JM1 genomic DNA; lanes 1 and 2, 5 µl of cDNAs obtained from RT reaction mixtures on 0.8 µg and 1.6 µg of JM1 total RNAs, respectively; lane (−), 1.6 µg of JM1 total RNAs. Lane L, DNA ladder. The sigma-70 promoter was predicted using the online tool BPROM (V. Solovyev and A. Salamov, p 61–78, *in* R. W. Li, ed., *Metagenomics and Its Applications in Agriculture, Biomedicine and Environmental Studies*, 2011). The transcription terminator was predicted using the ARNOLD web server (http://rna.igmors.u-psud.fr/toolbox/arnold/index.php) (D. Gautheret and A. Lambert, J Mol Biol 313:1003–1011, 2001; T. Macke et al., Nucleic Acids Res 29:4724–4735, 2001). Signal sequences were predicted with SignalP 4.1 (T. N. Petersen, S. Brunak, G. von Heijne, and H. Nielsen, Nat Methods 8:785–786, 2011). Download FIG S1, PDF file, 0.2 MB. Copyright © 2018 Mounier et al. 2018 Mounier et al. This content is distributed under the terms of the Creative Commons Attribution 4.0 International license.

**FIG 1:**
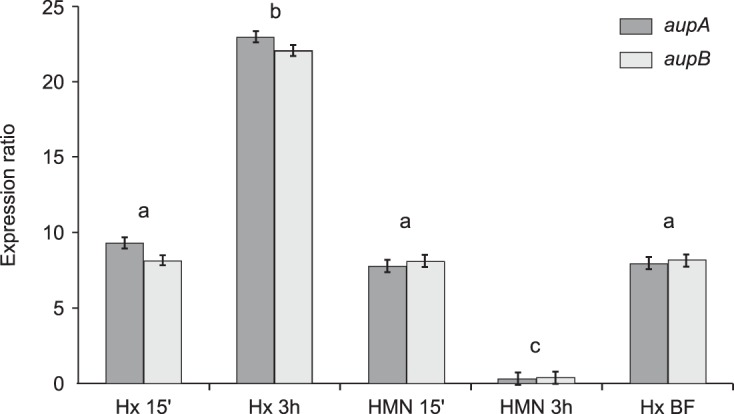
Relative expression levels of *aupA* and *aupB* genes measured by RT-qPCR. Expression levels in cells adhering to either *n-*hexadecane (Hx) or heptamethylnonane (HMN) for 15 min (15′) or 3 h or in biofilm cells growing in SSW plus 0.2% (vol/vol) *n-*hexadecane (Hx BF) are given as expression ratios in comparison to planktonic cells growing exponentially on acetate. The expression ratios were calculated using the threshold cycle (2^−ΔΔ*CT*^) method with the 16S rRNA gene as reference. The results are from at least 18 values collected from six technical replicates (two qPCRs realized from three distinct cDNA pools) obtained from three biological replicates. Significant differences were statistically assessed using one-way analysis of variance and Tukey’s honestly significant difference test, which provided three groups of growth conditions with similar expression ratios (a, b, and c).

The relative expression levels of the *aup* genes during biofilm growth were measured by reverse transcription-quantitative polymerase chain reaction (RT-qPCR) in comparison to their expression levels in planktonic cells growing on acetate. The relative expression levels of the two genes were first determined at initial stages of biofilm development, i.e., after 15 min and 3 h of adhesion to *n-*hexadecane– or HMN-water interfaces (Fig. 1). HMN is a branched nonmetabolizable hexadecane isomer that was used as a control for adhesion to a nonsubstrate hydrophobic compound. Cells were put into contact with alkane droplets obtained by emulsifying the alkane in synthetic seawater (SSW) by sonication. Contact with alkane-water interfaces induced a significant overexpression, compared to cells growing on acetate, of the *aup* genes as soon as 15 min of adhesion (9-fold for *aupA* and 8-fold for *aupB* on *n-*hexadecane; 8-fold for both genes on HMN). On *n*-hexadecane, the induction of both *aup* genes increased with time to reach a 23-fold and a 22-fold overexpression for *aupA* and *aupB*, respectively, after 3 h of adhesion. In contrast, after 3 h of adhesion to HMN, the expression of both genes returned to the reference level. This decrease in expression after adhesion to HMN could be due to energy and carbon exhaustion, as HMN is not metabolizable. In biofilm cells developing at the *n-*hexadecane–water interface, the two genes stayed significantly upregulated (8-fold for both *aup* genes) in comparison to cells growing exponentially on acetate (Fig. 1). We hypothesize that physiological cell heterogeneity that may occur in mature biofilms might explain this lower overexpression in comparison to 3 h of adhesion, with a proportion of biofilm cells putatively no longer overexpressing these two genes. These results hint toward a role of *aupA* and *aupB* in growth as a biofilm on alkanes.

### The *aupA* and *aupB* genes are involved in growth on alkanes.

To investigate the role of *aupA* and *aupB* in alkane assimilation, the mutations Δ*aupA*::*aphA*, Δ*aupB*::*aphA*, and Δ*aupAB*::*aphA* were generated in the strain JM1 by allelic exchange, giving the strains JM2, JM3 (here called the Δ*aupB* mutant), and JM4 (here called the Δ*aupAB* mutant), respectively. The Δ*aupA*::*aphA* mutation abolished the expression of the downstream gene *aupB* most likely by polar effect (Fig. S2). Production of the AupB protein in JM2 was restored by the introduction of a copy of *aupB* carried by a mini-Tn*7* transposon, giving the strain JM6 (here named the Δ*aupA* mutant) (see Text S1, Table S1, and Fig. S2).

10.1128/mBio.00520-18.1 TEXT S1  Supplemental materials and methods. Download TEXT S1, PDF file, 0.3 MB. Copyright © 2018 Mounier et al. 2018 Mounier et al. This content is distributed under the terms of the Creative Commons Attribution 4.0 International license.

10.1128/mBio.00520-18.3 FIG S2  Immunoblotting assays on Δ*aup* mutants and complemented strains. Total protein extracts from M. hydrocarbonoclasticus strains grown on SSW*-*acetate were analyzed by Western blotting using either anti-AupA or anti-AupB antiserum. JM1, wild-type strain; JM2, Δ*aupA*::*aphA* knockout mutant in JM1; JM3, Δ*aupB*::*aphA* knockout mutant in JM1; JM4, Δ*aupAB*::*aphA* knockout mutant in JM1; JM5, JM2 that carries wild-type *aupA* under its own promoter on a mini-Tn*7*T; JM6, JM2 that carries wild-type *aupB* under the PA_1/04/03_ promoter on a mini-Tn*7*T; JM7, JM2 that carries wild-type *aupAB* under its own promoter on a mini-Tn*7*T; JM8, JM3 that carries wild-type *aupB* under the PA_1/04/03_ promoter on a mini-Tn*7*T; JM9, JM4 that carries wild-type *aupAB* under its own promoter on a mini-Tn*7*T. Download FIG S2, PDF file, 0.1 MB. Copyright © 2018 Mounier et al. 2018 Mounier et al. This content is distributed under the terms of the Creative Commons Attribution 4.0 International license.

10.1128/mBio.00520-18.7 TABLE S1  Bacterial strains, plasmids, and primers. Download TABLE S1, PDF file, 0.8 MB. Copyright © 2018 Mounier et al. 2018 Mounier et al. This content is distributed under the terms of the Creative Commons Attribution 4.0 International license.

**FIG 2:**
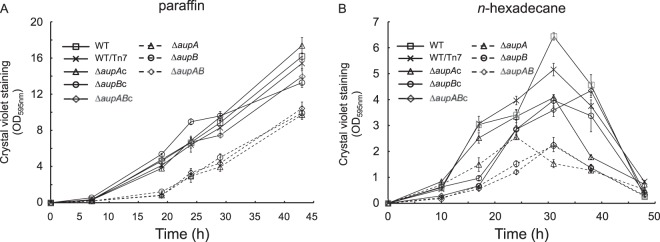
Kinetics of biofilm formation on alkanes for *aup* mutants. The quantification of biofilm formation on solid paraffin (A) and at the *n-*hexadecane–water interface (B) was assayed by crystal violet staining as described in Materials and Methods. Each value is from biological triplicates. On paraffin, biofilm mutant values were statistically (permutational *t* test) different from the wild type (WT) and complemented strains from their respective mutant at 19, 24, 29, and 43 h (*P* value ≤ 0.005). On *n*-hexadecane, biofilm mutant values were statistically different from the wild type and complemented strains from their respective mutant at 17, 24, 31, and 38 h (*P* value ≤ 0.02).

The kinetics of biofilm formation on solid paraffin (mixture of C_19_ to C_31_ alkanes) showed that all the Δ*aup* mutants formed a biofilm at a lower rate than the wild type during the first 20 h of growth. Rates of biofilm formation of the mutants and the wild type became similar after 20 h of growth. The biofilm biomass accumulated by the mutant strains at 43 h was about two-thirds of that of the wild type (Fig. 2A). All the Δ*aup* mutants showed a rate of biofilm formation lower than that of the wild-type strain on the liquid alkane *n-*hexadecane (Fig. 2B). Biofilm formation on paraffin was completely restored in JM7, JM8, and JM9 complemented strains (Table S1) (here called Δ*aupA*c, Δ*aupB*c, and Δ*aupAB*c, respectively), whereas, for an unknown reason, the complementation was only partial on *n-*hexadecane (Fig. 2B). Biofilm formation on *n*-alkanes with 12, 14, 20, and 21 carbon atoms was also affected in the Δ*aup* mutants (data not shown). Moreover, the amounts of *n-*hexadecane degraded in 26-h cultures of the Δ*aupA*, Δ*aupB*, or Δ*aupAB* mutant were smaller than in wild-type culture, showing a defect in *n-*hexadecane degradation in both mutant strains (Fig. S3). In contrast, the development of the mutant biofilms on hexadecanol (a fatty alcohol), palmitic acid (a fatty acid), hexadecyl hexadecanoate (a wax ester), and tripalmitin (a triglyceride) was not affected (Fig. S4). Moreover, the planktonic growth of the Δ*aup* mutants on water-soluble substrates (small organic acids, amino acids, and Tween 20) was indistinguishable from the wild type (data not shown). Thus, the growth phenotypes of the Δ*aup* mutants showed that the AupA and AupB proteins are involved in growth and/or biofilm formation on *n*-alkanes.

10.1128/mBio.00520-18.4 FIG S3  Hexadecane degraded in 20 h in biofilm cultures of M. hydrocarbonoclasticus wild-type and mutant strains: WT (wild-type JM1), Δ*aupA* (JM6), Δ*aupB* (JM3), Δ*aupAB* (JM4), Δ*aupA*c (JM7), Δ*aupB*c (JM8), and Δ*aupAB*c (JM9) strains. Error bars represent the standard error from three biological replicates. (a) Values obtained with the mutants (Δ*aupA*, Δ*aupB*, and Δ*aupAB*) were statistically different with respect to the wild type (*P* value ≤ 0.03). (b) Values of the complemented strains (Δ*aupA*c, Δ*aupB*c, and Δ*aupAB*c) were statistically significantly different from their respective mutants (*P* value ≤ 0.04) and not statistically significantly different from the wild-type JM1 (*P* value ≥ 0.2). Download FIG S3, PDF file, 0.1 MB. Copyright © 2018 Mounier et al. 2018 Mounier et al. This content is distributed under the terms of the Creative Commons Attribution 4.0 International license.

10.1128/mBio.00520-18.5 FIG S4  Kinetics of biofilm formation on hydrophobic compounds. Biofilms were quantified by crystal violet staining. Error bars represent the standard error from three biological replicates. Download FIG S4, PDF file, 0.2 MB. Copyright © 2018 Mounier et al. 2018 Mounier et al. This content is distributed under the terms of the Creative Commons Attribution 4.0 International license.

### Δ*aupA*, Δ*aupB*, and Δ*aupAB* mutants are impaired in the assimilation of *n-*hexadecane solubilized in surfactant micelles.

**FIG 3:**
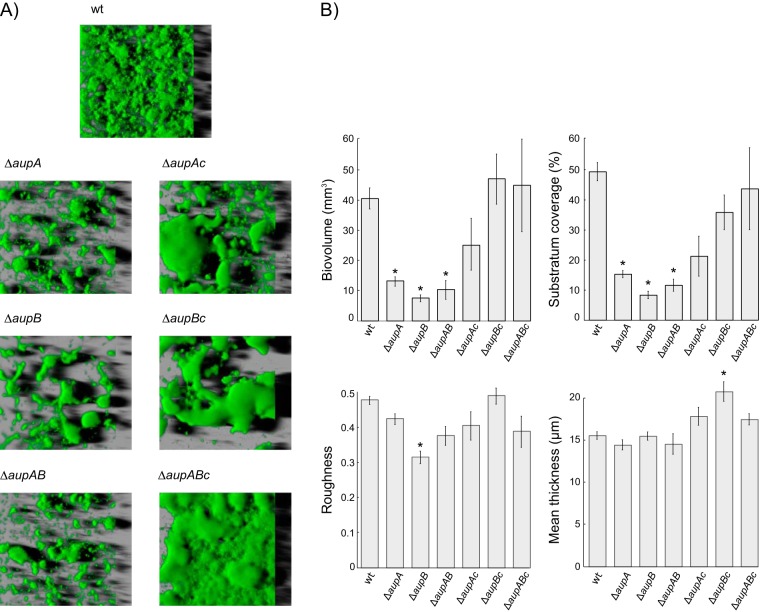
Confocal microscopy imaging of wild-type and mutant M. hydrocarbonoclasticus biofilms grown on alkanes. (A) Three-dimensional images of 24-h biofilms grown on paraffin and stained with Syto 9 (green). (B) Quantitative analysis of biofilm architectures. Total biovolume, substratum coverage, roughness, and mean thickness were determined for 24-h biofilms on paraffin. Results are from triplicate measurements from two independent experiments. Asterisks indicate values that are statistically different from the wild type (wt) (*P* value < 0.05).

The growth deficiency of the Δ*aup* mutants on *n*-alkanes could be due either to a defect in the structural or functional organization of the biofilm, which would impair the assimilation of alkanes, or to a deficiency in alkane assimilation (i.e., uptake, transport, or metabolism), which would affect the growth and hence the development of the biofilm. In an attempt to discriminate between these two possibilities, the structures of the mutant and wild-type biofilms grown for 24 h on paraffin were investigated by confocal microscopy (Fig. 3). The lower biovolume and surface coverage of the mutant biofilms indicated that *aup* mutations cause a deficiency in biomass production and in the ability to colonize the surface, which is consistent with the results obtained with the crystal violet biofilm assay (Fig. 2A). However, the mean thicknesses and the roughnesses of mutants and wild-type biofilms were similar, which is indicative of a similar spatial heterogeneity. Thus, although mutant biofilms developed to a lesser extent, they exhibited an overall structure similar to that of the wild type.

**FIG 4:**
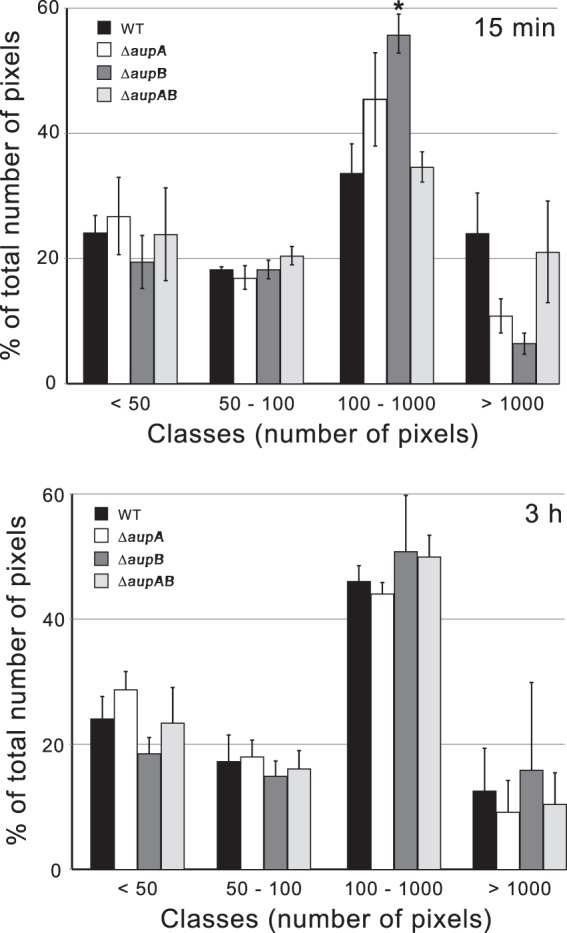
Measurement of cell adhesion to *n-*hexadecane droplets. Images of cells incubated with emulsified *n-*hexadecane for 15 min and 3 h were taken by confocal microscopy. Adhered cells formed clusters around *n-*hexadecane droplets. The number and the size distribution of cell clusters therefore reflect the extent of adhesion to *n-*hexadecane. Single bacteria or clusters of bacteria were sorted into four classes according to the number of pixels that they represented on the images: <50, 50 to 100, 100 to 1,000, and >1,000 pixels. The number of pixels in each class is reported as a percentage of the total number of pixels in the image. The quantifications were performed from four different images. The asterisk indicates a statistical difference from the wild type (*P* value < 0.05).

Cell adhesion to *n-*hexadecane droplets was next evaluated through the analysis of confocal microscopy images. For that purpose, bacterial cells were mixed with an emulsion of *n*-hexadecane in SSW. As adherent cells formed clusters or aggregates around *n-*hexadecane droplets, the extent of cell aggregation was used to evaluate the adhesion to *n-*hexadecane droplets. Figure 4 shows that the Δ*aup* mutants and the wild-type strain exhibited very similar cell cluster distributions, thus indicating that the Δ*aup* mutants were not impaired in adhesion to the alkane-water interface. These observations suggest that *aupA* and *aupB* are not directly involved in biofilm development but should rather act in the alkane assimilation process.

**FIG 5:**
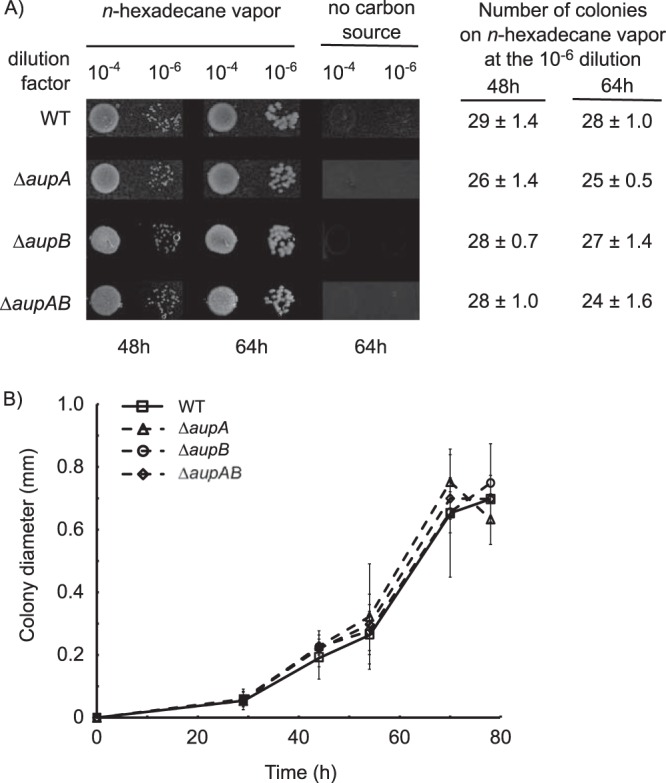
Growth phenotypes of wild type and *aup* mutants on agar plates in vapor of *n*-hexadecane. Cells were cultivated in SSW-20 mM acetate until the stationary growth phase (OD_600_, ~0.7) and washed twice with SSW without carbon source. Ten-microliter-drops of serial dilutions (dilution factor) were plated on SSW agar plates. The headspaces of the plates were saturated with *n*-hexadecane by gluing a piece of Whatman paper presoaked with *n-*hexadecane under the lid. Plates were sealed and incubated at 30°C for 48 and 64 h. (A) Pictures of colonies and numbers of colonies estimated from cultures diluted 1 × 10^6^ times. The number and the size of colonies formed by the mutants were not statistically different with respect to the wild type (*P* value ≥ 0.625). (B) Size of colonies growing on hexadecane vapor plates. Pictures of colonies were taken at different times, and the colony diameter was measured using the ImageJ software. Values obtained with the mutants were not statistically different with respect to the wild type (*P* value ≥ 0.06).

**FIG 6:**
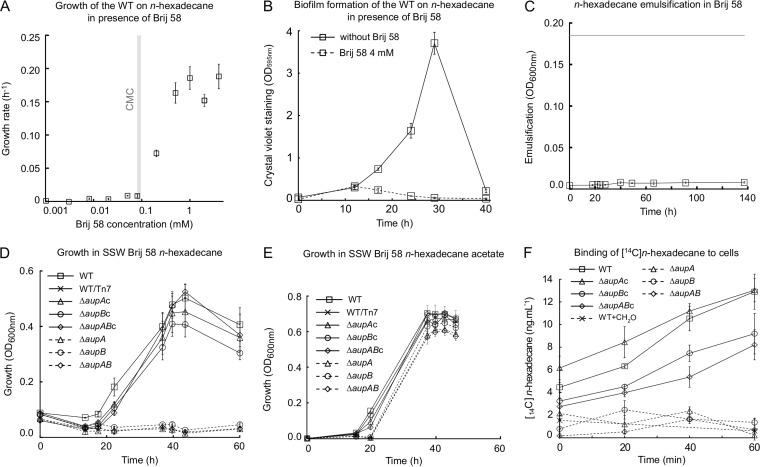
Growth on micelle-solubilized *n-*hexadecane. (A) Relationship between bacterial growth rate on *n-*hexadecane and Brij 58 concentration. The vertical gray bar marks the Brij 58 CMC (0.077 mM). All cultures were done in triplicate. (B) Crystal violet biofilm assay of M. hydrocarbonoclasticus JM1 growing on *n-*hexadecane in the presence or absence of 4 mM Brij 58. Assays were done in triplicate. (C) Emulsification assay of *n-*hexadecane in SSW-Brij 58. Emulsification assays were performed in triplicate in glass tubes containing 5 ml SSW, 4 mM Brij 58, and 0.2% (vol/vol) *n-*hexadecane incubated at 30°C and 200 rpm. Emulsification of *n*-hexadecane into the aqueous phase was monitored by measuring the OD_600_. The gray line indicates the absorbance of *n*-hexadecane emulsified in SSW-4 mM Brij 58 by sonication. (D) Growth kinetics of the *aup* mutants on *n-*hexadecane in the presence of 4 mM Brij 58. The cultures were done in triplicate, and growth was followed by measuring OD_600_. Mutant values were statistically different from the wild type and the complemented strains at 23, 38, 41, 45, and 62 h (*P* value ≤ 0.05). (E) Growth curves in SSW–4 mM Brij 58–0.2% (vol/vol) *n*-hexadecane and 20 mM acetate. Each value is the mean of biological triplicates. Values from the mutants and the complemented strains were not statistically different from the wild type (*P* value ≥ 0.095). (F) Incorporation of [^14^C]*n*-hexadecane into M. hydrocarbonoclasticus cells. Measurements are from three to nine biological replicates. Values obtained with the mutants and the formaldehyde-killed cells were statistically different with respect to the wild type (*P* value ≤ 0.004). Values of the Δ*aupA*c and Δ*aupB*c complemented strains were not statistically significantly different from the wild type (*P* value ≥ 0.1). Values of the Δ*aupAB*c complemented strain were statistically different from both the wild type and the Δ*aupAB* mutant (*P* value ≤ 0.05).

To confirm this interpretation, we attempted to uncouple alkane assimilation and biofilm formation. Biofilm formation occurs in cultures where the substrate and the cells are partitioned into two different phases. We therefore provided *n*-hexadecane in the same phase as the cells. This could be achieved in two ways: by growing M. hydrocarbonoclasticus on agar plates incubated in vapor of *n-*hexadecane and by pseudosolubilization of the alkane in the aqueous phase in surfactant micelles. The wild-type and Δ*aup* mutant strains were first plated on SSW agar with no carbon source and incubated with *n-*hexadecane supplied in the vapor phase. The number and the size of colonies were alike in the wild type and the mutants, indicating that *aupA* and *aupB* are not required for growth when *n-*hexadecane is provided in the gas phase (Fig. 5). To test the ability of M. hydrocarbonoclasticus to assimilate pseudosolubilized *n*-hexadecane in surfactant micelles, the wild-type strain was incubated in SSW plus *n*-hexadecane in the presence of various concentrations of Brij 58, a surfactant that is neither toxic nor used by M. hydrocarbonoclasticus as a substrate (Fig. S5). At Brij 58 concentrations below the critical micelle concentration (CMC), i.e., 0.077 mM (26), growth on *n-*hexadecane was completely inhibited (Fig. 6A). In contrast, when Brij 58 concentrations exceeded the CMC, growth was observed with a maximum rate for Brij 58 concentration above 6.5 times the CMC. The fact that the CMC is a threshold concentration above which growth is enabled indicates that the micellar form of the surfactant is required for the utilization of *n-*hexadecane. Furthermore, in the presence of Brij 58, no biofilm at the *n-*hexadecane–water interface and no emulsification of the alkane were observed (Fig. 6B and C). We concluded that, at concentrations below the CMC, Brij 58 inhibited growth on *n-*hexadecane most likely by preventing biofilm formation, whereas at concentrations above the CMC, micelle formation allowed M. hydrocarbonoclasticus to use *n-*hexadecane partitioned into them, with no requirement for biofilm formation. Growth on micelle-solubilized *n*-hexadecane exhibited a pseudolinear kinetic, suggesting that transfer of *n*-hexadecane from micelles to the cell is limiting. In contrast, Δ*aup* mutants did not grow at all on *n-*hexadecane plus Brij 58 at 52 times the CMC while the complemented strains grew as well as the wild type (Fig. 6D). The mutants were able to grow in SSW–Brij 58–*n*-hexadecane in the presence of acetate (Fig. 6E), indicating the nontoxicity of *n*-hexadecane solubilized in Brij 58 toward the mutants. The ability of the mutants to grow on *n*-hexadecane vapor but not on micelle-solubilized *n*-hexadecane points to a role of AupAB in the uptake of *n*-hexadecane.

10.1128/mBio.00520-18.6 FIG S5  Kinetics of planktonic growth of M. hydrocarbonoclasticus strains on acetate in the presence of Brij 58. (A) Kinetics of planktonic growth of the wild-type JM1 strain on SSW-20 mM acetate or on SSW-20 mM acetate in the presence of 4 mM Brij 58. (B) Kinetics of JM1 and mutant strains (Δ*aupA*, Δ*aupB*, and Δ*aupAB*) on SSW-20 mM acetate in the presence of 4 mM Brij 58. Measurements were done on biological triplicates. Download FIG S5, PDF file, 0.1 MB. Copyright © 2018 Mounier et al. 2018 Mounier et al. This content is distributed under the terms of the Creative Commons Attribution 4.0 International license.

To test this hypothesis, the amount of [^14^C]*n*-hexadecane that was bound to or incorporated into resting wild-type and *aup* mutant cells was determined (Fig. 6F). Prior to measurements, cells were preincubated for 30 min in the presence of [^14^C]*n*-hexadecane solubilized in Brij 58 micelles to ensure induction of *aup* genes. Then, protein synthesis was stopped by chloramphenicol addition to keep the amount of Aup proteins constant during the assay. The wild type exhibited an increase of cell-associated [^14^C]*n*-hexadecane over the 60 min of incubation, while [^14^C]*n*-hexadecane association with formaldehyde-killed cells was very low and did not increase over time. The nonnull value at time zero was attributed to the association of [^14^C]*n*-hexadecane with cells during the 30-min preincubation. Levels of [^14^C]*n*-hexadecane associated with *aup* mutant cells were indistinguishable from that of formaldehyde-killed cells. Although this experiment cannot distinguish between binding and actual incorporation (i.e., transport into the cell), it indicates that micellar *n*-hexadecane is at least rapidly associated with wild-type cells and that this does not occur in the *aup* mutants. This result demonstrates that Aup proteins are involved in the binding or the incorporation of *n*-hexadecane and hence support a role in uptake.

### AupA and AupB are outer and inner membrane proteins interacting together.

**FIG 7:**
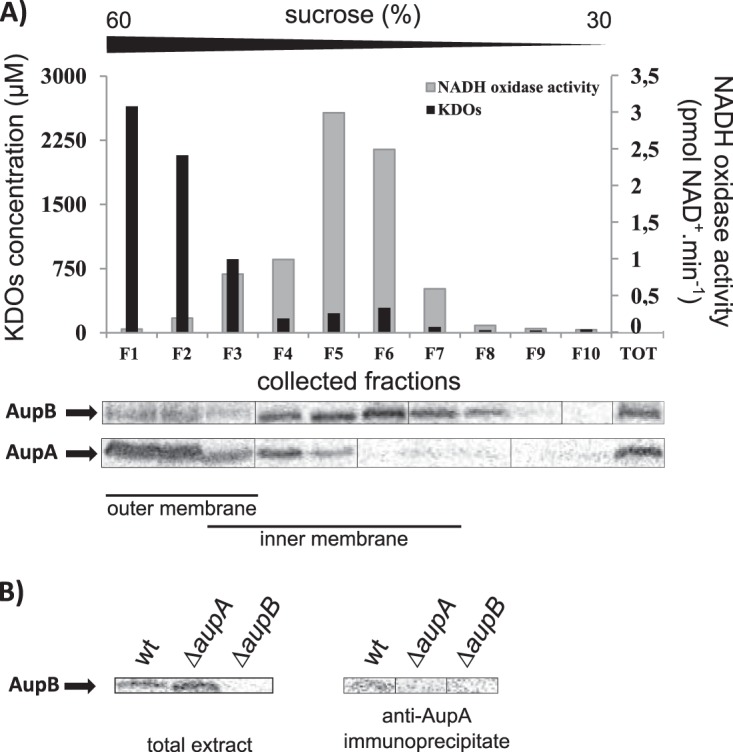
Subcellular localization and coimmunoprecipitation of AupA and AupB. (A) Subcellular localization of AupA and AupB. Total membranes from JM1 cells obtained from an exponential culture grown on SSW-20 mM acetate were separated on a discontinuous sedimentation sucrose gradient. Membrane fractions were collected from the bottom of the tube. The sucrose gradient (from 30% to 60%) is indicated above the graph. The immunoblotting assays were performed with anti-AupA or anti-AupB antisera on all fractions collected (F1 to F10) and on total membrane extract (TOT) loaded on the gradient. NADH oxidase activity (gray) and KDOs (black) were used as markers of the inner and outer membranes, respectively. (B) Coimmunoprecipitation of AupA and AupB. Immunoprecipitations were carried out on extracts prepared from cells in exponential growth phase (OD_600_, ~0.5) on SSW-20 mM acetate. Anti-AupA antiserum was added to cell lysates, and the immune complex was recovered by affinity to protein A-Sepharose beads. See Text S1 for more details. Anti-AupB immunoblots of total extracts and immunoprecipitated fractions are shown.

The involvement of AupA and AupB in alkane uptake implies that they localize in the cell envelopes. To confirm this, we analyzed by immunoblotting with anti-AupA and anti-AupB antisera a membrane extract separated by sucrose gradient sedimentation. As shown in Fig. 7A, AupA was detected in the high-density fractions containing 2-keto-3-deoxyoctonates (KDOs), which are components of the lipopolysaccharides used as markers of the outer membrane. Most of the AupB protein cosedimented with the NADH oxidase activity used as a marker of the inner membrane. These results demonstrate that AupA and AupB are associated with the outer and inner membranes, respectively. Moreover, a small amount of AupB cofractionated with AupA in the KDO fractions, suggesting that these two proteins could form a complex. This was indeed confirmed by the coimmunoprecipitation of AupB with the anti-AupA antiserum (Fig. 7B).

### *aupA* and *aupB* are conserved among marine HCB.

**FIG 8:**
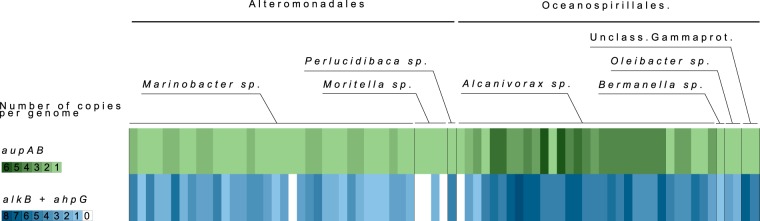
Phylogenetic distribution of *aupA* and *aupB* genes and cooccurrence of alkane hydroxylase genes. All genomes from the IMG database containing *aupA* and *aupB* homologues are displayed in columns and taxonomically classified. Numbers of *aupAB* genes and *ahpG* plus *alkB* in each genome are displayed in lines and color coded.

In order to obtain insights into the ecophysiological role of *aupA* and *aupB*, their taxonomic distribution and their genetic organization were explored in bacterial genomes. These two genes were found in 76 bacteria for which genome sequences were available in the IGM/M (Integrated Microbial Genomes & Microbiomes) database. The genomes containing *aupA* also contain *aupB* and vice versa, except for the genome of *Alcanivorax* sp. strain JRC, which contains only *aupB*. The *aupAB*-containing genomes belong to the following genera: *Alcanivorax* (31 genomes), *Bermanella* (1 genome), and *Oleibacter* (2 genomes) of the order *Oceanospirillales*; *Marinobacter* (27 genomes), *Moritella* (4 genomes), and *Perlucidibaca* (1 genome) of the order *Alteromonadales*; and two unclassified *Gammaproteobacteria* (Fig. 8; Table S2). All these strains, except HdN1, have been isolated from marine or saline environments. Moreover, 10 of them have been isolated from hydrocarbon-contaminated environments and 17 strains were shown to use alkanes as a carbon source. All but one (*Alcanivorax* sp. JRC) strains presented an *aupA-aupB* organization similar to the operon of M. hydrocarbonoclasticus SP17, with both CDS in the same orientation and separated by a small intergenic region. In 36 strains, tandems of *aupA* and *aupB* were present in multiple nonidentical copies, the maximum being six copies in *Alcanivorax* sp. strain 43B_GOM-46m and in *Alcanivorax* sp. strain DG881. Some strains also contained additional isolated copies of either *aupA* or *aupB* (Fig. 8; Table S2). We next examined the cooccurrence of the alkane catabolism genes *alkB* and *ahpG* with the *aupAB* tandem. Most of the *aupAB*-containing genomes (70 out of 75) exhibited at least one alkane catabolism gene (Fig. 8; Table S2). The cooccurrence of *aupAB* with alkane degradation genes, together with the very narrow taxonomic distribution of the *aup* genes, including almost exclusively strains belonging to genera of which members are recognized as marine hydrocarbonoclastic bacteria, suggests a niche-specific role for *aupA* and *aupB* in alkane assimilation in the marine environment.

10.1128/mBio.00520-18.8 TABLE S2  Phylogenetic distribution of *aupA* and *aupB* genes and cooccurrence of alkane hydroxylase genes. Genomes coding for AupA or AupB homologues were searched in the set of complete genomes of isolated strains of the IMG database using BLASTP. Criteria for positive hits were more than 30% identity and more than 80% of the query sequence aligned. *, possible sequencing errors or mutations resulting in fragmented proteins. Uncl, unclassified; Unpub, unpublished data; NA, not available; *alkB*, alkane 1-monooxygenase gene; *ahpG*, cytochrome P450 hydroxylase gene. Download TABLE S2, PDF file, 0.7 MB. Copyright © 2018 Mounier et al. 2018 Mounier et al. This content is distributed under the terms of the Creative Commons Attribution 4.0 International license.

## DISCUSSION

### AupA and AupB are required for the uptake of *n-*hexadecane solubilized in surfactant micelles.

In this study, we undertook the functional characterization of the *aupA* and *aupB* genes, which form an operon inducible by metabolizable alkanes. The overexpression of *aupA* and *aupB* after only 15 min of contact with *n*-hexadecane droplets suggested a role in alkane utilization. This was further supported by growth and degradation tests showing that Δ*aup* mutants were affected in the growth on alkanes. Moreover, it is very likely that both genes play a role in the same molecular process since their deletion resulted in identical phenotypes and a complex between AupA and AupB was detected.

In liquid cultures in minimum mineral medium where alkanes form a second liquid or solid phase, growth of M. hydrocarbonoclasticus SP17 occurs exclusively through the formation of a biofilm at alkane-water interfaces (13). Thereby, the reduced growth on alkane of the *aup* mutants could be due to a deficiency in alkane assimilation, a defect in biofilm development, or an increased sensitivity to alkanes. An increased sensitivity of the mutants to *n*-hexadecane can be ruled out since *aup* mutants were able to grow at the same rate as the wild type on *n*-hexadecane vapor or in SSW–Brij 58–*n*-hexadecane supplemented with acetate. A defect in the development of the biofilm is unlikely since the *aup* mutants were not affected in adhesion to *n-*hexadecane and since the overall structure of their biofilms developing on paraffin was not disorganized. These results point to a role in alkane assimilation of AupA and AupB. The assimilation process can be divided into two steps: (i) the uptake phase, during which alkanes are captured from the extracellular environment and transported across the cell envelope, and (ii) the metabolic phase, during which alkanes are transformed into metabolites to feed biosynthesis or energy production pathways. The growth of the *aup* mutants on *n*-hexadecane vapor showed that they were still able to degrade *n*-hexadecane at a similar rate as the wild type, thus pointing to a role of Aup proteins in the alkane uptake process rather than in alkane metabolism. The involvement of Aup proteins in alkane uptake is also strongly supported by the fact that the phase in which the alkanes were provided in the culture impacted profoundly the extent of the growth defect of *aup* mutants. The growth of *aup* mutants was not affected when alkanes were provided to cells in the vapor phase at the agar-air interface. In contrast, when the alkanes were available to the cells as an interface with the aqueous phase, growth of the *aup* mutants was impaired but not completely abolished. Mutants did not grow at all when *n*-hexadecane was solubilized in surfactant micelles. The *aup* mutants lost their ability to bind to or incorporate [^14^C]*n*-hexadecane solubilized in micelles of Brij 58. This demonstrates that Aup proteins are involved in binding to or incorporation into the cells of micellar *n*-hexadecane, which argues strongly in favor of a role of AupA and AupB in micellar alkane uptake.

AupA exhibits 21.1% identity and 53.9% similarity to the long-chain fatty acid transporter FadL (24). The homology of AupA with this transporter hints at a role of AupA as an outer membrane channel involved in alkane transport into the cell. According to the model of lateral diffusion, FadL-like transporters release their substrates in the outer membrane. The substrates must then reach and eventually cross the cytoplasmic membrane through desorption from the outer membrane and diffusion across the periplasm and the cytoplasmic membrane. Considering that long-chain fatty acids were shown to readily desorb from phospholipid bilayers, it was proposed that desorption from the inner leaflet of the outer membrane would be driven by simple action mass (23, 27). However, long-chain alkanes are about a thousand times less soluble than the corresponding long-chain fatty acids. It can be expected that long-chain alkane desorption from the outer membrane would be much slower and therefore would require a protein that facilitates their transfer from the outer membrane toward the cytoplasmic membrane, which contains the alkane monooxygenase, the first enzyme of the alkane degradation pathway. Given the dual localization of AupB, associated both with the cytoplasmic membrane and with AupA in the outer membrane, it is tempting to propose that AupB could facilitate the transfer of alkanes from the outer membrane to the cytoplasmic membrane.

Our results provide the first evidence for proteins involved in assimilation of micelle-solubilized compounds. Micellar uptake of HOCs has been reported in several bacterial strains with different surfactants. Guha and Jaffé (28, 29) developed a theory to explain the biodegradation of micellar HOCs that involves a transfer of the HOCs from the micelles present in the water phase to a hemimicellar layer formed around the cells. In a last step, the HOCs would be transferred from the hemimicelles into the cell. Recently, direct evidence of hydrocarbon partitioning into surfactant hemimicelles on the bacterial cell surface was provided (30).

### Role of AupA and AupB in alkane uptake within biofilms.

The involvement of AupAB in micellar uptake of alkane and the defect in biofilm growth on alkane of the *aup* mutants suggest the existence of a micellar uptake pathway for the assimilation of alkanes in the biofilm. Such a pathway implies the presence of micelle-forming compounds within the biofilm. Micelles can be formed by biosurfactants, usually glycolipids or peptidolipids as well as proteins (7, 31). Although the production of small biosurfactant molecules by M. hydrocarbonoclasticus has never been detected, the release of high-molecular-weight surface-active compounds when cells adhere to the *n-*hexadecane–water interface was reported previously (12). More recently, we showed that the extracellular matrix of biofilms growing on *n*-hexadecane is mainly composed of proteins whose surface-active properties have been documented. Moreover, an unidentified extracellular protein secreted through the type II secretion system was shown to be essential for alkane assimilation through biofilm formation and is most likely involved in the mass transfer or capture of alkanes (32). It is therefore conceivable that the AupAB-mediated uptake uses proteins present in the extracellular matrix of the biofilm as micelles.

In aquatic environments, combining micellar uptake with biofilm formation appears necessary. The extracellular matrix of biofilm creates an environment that could retain surface-active compounds and allow their accumulation to a concentration above their CMC to form micelles. However, we acknowledge that in the absence of data on the action mechanism of AupAB within the biofilm, it is difficult to draw any firm conclusion about their participation in a micellar uptake pathway within biofilms and that the involvement of AupAB in nonmicellar pathways in biofilm is still possible.

### Existence of *aupAB*-independent alkane uptake pathways.

The Δ*aup* mutants were still able to grow, although at a reduced rate, on liquid and solid alkanes. Moreover, their growth rate on *n-*hexadecane supplied in the vapor phase was unchanged. This indicates that M. hydrocarbonoclasticus SP17 possesses other alkane uptake proteins. One of these could be the AlkL protein, which is encoded by *MARHY2842* in the genome of M. hydrocarbonoclasticus SP17. AlkL of Pseudomonas putida GPo1 has been shown to play a role in alkane mass transfer into cells (20, 21). The existence of other, not-yet-discovered uptake systems is also possible. The redundancy of uptake systems would allow high rates of assimilation of alkanes and could explain at least partly the high growth rate observed on these nearly insoluble substrates.

### The *aup* operon is widespread in marine HCB and frequently present in multiple copies.

Phylogenetic analyses revealed that the *aup* genes are found only in a limited number of genomes. Most of these genomes (95%) are from marine bacteria containing at least one copy of a gene encoding an alkane hydroxylase. The cooccurrence of the *aup* genes with alkane degradation genes is consistent with their proposed role in alkane uptake. The *aup* genes were found only across a few genera of the *Oceanospirillales* (*Alcanivorax*, *Bermanella*, and *Oleibacter*) and of the *Alteromonadales* (*Marinobacter*, *Moritella*, and *Perlucidibaca*). Representatives of these two orders have been recognized as being among the most important degraders in hydrocarbon-contaminated seawaters (2, 3). Recently, during the Deepwater Horizon oil spill, a deep-sea oil plume was shown to be strongly enriched in taxa of the *Oceanospirillales* order and to a lesser extent of the *Marinobacter* genus (33). The fact that strains carrying *aup* homologues are strongly represented in oil-degrading isolates from samples impacted by the Deepwater Horizon spill is consistent with a role of these genes in adaptation to oil contamination (34). A role for the *aup* genes in the response to oil contamination is also supported by gene expression data showing that *aupA* overexpression occurred in response to the presence of hydrocarbons under laboratory conditions (Fig. 1) as well as in complex microbial communities of a crude oil-amended microcosm (35).

The *aup* operon is often present in multiple copies in genomes, especially in the *Alcanivorax* genus, where some species carry six copies of this operon. Multiple copies of genes can occur as an evolutionary response in bacteria that have been exposed to selective pressures such as carbon starvation (36). The presence of multiple copies of a gene can be a way to increase the amount of the gene product, to have different genes regulated by different conditions, or to create functional divergence (37, 38). If the selective pressure is removed, the extra copies of genes are expected to be rapidly lost. In order to be maintained, multiple copies of a gene must therefore represent a selective advantage. The occurrence of the *aup* genes restricted to the group of marine HCB and their presence in high copy number in certain representatives of this group suggest that *aupAB* might be one attribute of the remarkable adaptation of HCB that allows them to take advantage of the hydrocarbons as carbon and energy sources and to occupy a peculiar trophic niche.

## MATERIALS AND METHODS

### Bacterial strains and culture conditions.

The bacterial strains and plasmids constructed and used in this study are listed in Table S1 in the supplemental material. JM1, a spontaneous streptomycin-resistant mutant (mutation *rpsLK58T*) isolated from the original isolate SP17 ATCC 49840 (39), was used as a parental strain to construct all M. hydrocarbonoclasticus derivatives and is referred to as the wild type in this study. Additional information is available in Text S1.

### Construction of knockout mutants by allelic exchange.

Mutant constructions by allelic exchange were performed in JM1 using the suicide vector pKAS32 (40) or its derivative pKOMKm (see Text S1 and Table S1). The mutant alleles of the *aup* genes were generated by replacing 683 bp of the *aupA* CDS and 1,920 bp of the *aupB* CDS with a 1,755-bp kanamycin resistance cassette (harboring the *aphA* gene) to give the Δ*aupA*::*aphA* and Δ*aupB*::*aphA* alleles, respectively. The operon mutant was obtained by the replacement of the whole *aupA* and *aupB* CDS by the kanamycin resistance cassette to give the Δ*aupAB*::*aphA* allele. Suicide vectors were introduced in JM1 by conjugation, and correct insertions of the mutant alleles were verified by PCR. For complementation experiments, the *aupA* and *aupAB* genes were cloned into pUC18T-mini-Tn*7*T-Gm (41) under the control of their native promoter while *aupB* was cloned under the control of the constitutive P_A1/04/03_ promoter. All the constructs were verified by sequencing. Details of mutant constructions are given in Text S1 and in Table S1. Insertion of mini-Tn*7*T derivatives in the M. hydrocarbonoclasticus strains was performed by four-parental mating conjugation as described by Choi et al. (41) with modifications described in Text S1. Insertions of the mini-Tn*7*T at the correct locus (one single *att*Tn*7* integration site in M. hydrocarbonoclasticus) were verified by PCR.

### Cell adhesion to HOCs.

The preparation of M. hydrocarbonoclasticus cells adherent to HOCs was carried out as described by Vaysse and Grimaud (42) with minor modifications: 4 ml of a culture in exponential growth phase (optical density at 600 nm [OD_600_] of ~0.5) on SSW plus 20 mM acetate was mixed with 4 ml of SSW-HOC (0.02%, vol/vol) emulsified by sonication, incubated under shaking at 50 rpm for 15 min or 3 h at 30°C in 50-ml BD Falcon tubes, and centrifuged at 15,000 × *g* for 15 min at 20°C. Cells bound to HOCs formed a gel-like phase on the top of the tube and were collected to be used directly for confocal microscopy imaging or stored at −80°C for RNA extraction.

### Growth kinetics with Brij 58.

Precultures of M. hydrocarbonoclasticus were grown in SSW supplemented with 4 mM Brij 58 (SSW-Brij 58) plus 20 mM acetate until the stationary growth phase (OD_600_, ~0.7). Cells were then collected by centrifugation for 5 min at 8,000 rpm, washed twice with SSW-Brij 58, and resuspended in the same volume of SSW-Brij 58. For assays with acetate as the substrate, the cell suspensions were diluted 150-fold in SSW-Brij 58 plus 20 mM acetate and incubated at 200 rpm at 30°C in glass tubes. For assays with *n-*hexadecane, the cell suspensions were diluted to an OD_600_ of ~0.1 in SSW-Brij 58 containing 0.2% (vol/vol) *n*-hexadecane and further incubated at 50 rpm and 30°C in Erlenmeyer flasks up to an OD_600_ of ~0.7. Cells were recovered by centrifugation, washed with SSW-Brij 58, and resuspended to an OD_600_ of ~0.05 in SSW-Brij 58 containing 0.2% (vol/vol) *n*-hexadecane. All SSW-Brij 58 media containing *n*-hexadecane were prepared 48 h in advance to allow for *n*-hexadecane solubilization in Brij 58 micelles. Cells were finally incubated at 50 rpm and 30°C. All cultures were done in triplicate, and growth kinetics were followed by measuring the OD_600_.

### Biofilm assays.

Biofilm formation on solid HOCs was assayed in 24-well polystyrene microplates (Evergreen Scientific). The bottoms of the wells were coated by melting 0.2 g of HOC per well for 1 h at 90°C and left at room temperature until HOC solidification. Cells in exponential growth phase on SSW-acetate were harvested by centrifugation at 10,000 × *g* for 15 min and resuspended to a final OD_600_ of 0.1 in SSW without any carbon source. A 1.5-ml amount of the cell suspension was then added to each HOC-coated well, and the microplate was incubated at 30°C and 100 rpm. The culture medium was then carefully withdrawn, and cells adhering to the solid HOC were stained for 3 min with 0.4 ml of 1% (wt/vol) crystal violet. After two washes with deionized water, crystal violet was extracted with 0.75 ml of an aqueous solution containing 10% (vol/vol) acetic acid and 50% (vol/vol) ethanol, and the absorbance was read at 595 nm. All measurements were done in quadruplicate. For liquid HOCs, 10 ml of a cell suspension in SSW prepared as described above was distributed in glass serum flasks (50 ml) and HOC was added at 0.2% (vol/vol). Cultures were incubated at 30°C and 50 rpm. Biofilms were recovered by filtration through a nylon membrane with a porosity of 31 µm (Nitex), stained for 3 min with 0.3 ml of 1% (wt/vol) crystal violet, and washed twice with 10 ml of deionized water. Crystal violet was extracted and quantified as described above. All measurements were done in triplicate.

### Incorporation of *n-*hexadecane into cells.

Cells in the exponential phase of growth (OD_600_ of 0.3 to 0.5) in SSW-20 mM acetate were collected by centrifugation at 4,500 × *g* for 15 min at room temperature and resuspended at an OD_600_ of 1 in SSW containing 4 mM Brij 58. The cell suspension was diluted 5-fold in SSW-20 mM acetate plus 4 mM Brij 58 containing 0.1% (vol/vol) [^14^C]*n*-hexadecane (5 µCi ⋅ ml^−1^) prepared 48 h in advance to allow for *n*-hexadecane solubilization in Brij 58 micelles. Cells were incubated for 30 min at 30°C, and then protein synthesis was stopped with chloramphenicol at 34 µg ⋅ ml^−1^. Association of [^14^C]*n*-hexadecane with cells was measured from this time by sampling 1 ml of the cell suspension at regular intervals over 60 min. Cells were fixed with 1% formaldehyde and harvested by centrifugation at 20,000 × *g* for 10 min at room temperature. The pellet was resuspended in 1 ml of SSW-4 mM Brij 58 by vortexing for 1 min and centrifuged at 20,000 × *g* for 10 min at room temperature, and the cell pellet was resuspended in 100 µl of SSW-4 mM Brij 58 and transferred in scintillation vials containing 5 ml of ACS scintillation liquid (GE Healthcare) Radioactivity was measured with a scintillation counter (LS 6500; Beckman).

### Membrane fractionation.

Inner and outer membranes were separated using sucrose gradient centrifugation. In total, 2 × 10^11^ cells growing exponentially (OD_600_ of ~0.5) on SSW plus 20 mM acetate were harvested by centrifugation at 20,000 × *g* for 15 min at 4°C, resuspended in 2 ml of lysis buffer (10 mM Tris-HCl, pH 7.4, 20% [vol/vol] sucrose, 10 µg/ml RNase A, 10 µg/ml DNase I, 8.6 mg/ml protease inhibitor cocktail), and lysed three times through an ice-chilled French press at 18,000 lb/in^2^. Cell debris was removed by centrifugation at 15,000 × *g* for 30 min at 4°C, and total membranes were harvested by ultracentrifugation at 100,000 × *g* for 60 min at 4°C (rotor 18426; Kontron Instruments). The membrane pellet was resuspended in 500 µl of lysis buffer and loaded on the top of a discontinuous sucrose gradient consisting of 600 µl of 30%, 35%, 40%, 45%, 50%, 55%, and 60% (wt/vol) sucrose solutions prepared in 10 mM Tris-HCl, pH 7.4. The gradient was centrifuged at 90,000 × *g* for 72 h at 4°C (rotor SW50.1; Beckman), and 400-µl fractions were collected from the bottom. Inner and outer membrane-containing fractions were identified by measuring NADH oxidase activity and KDO concentration, respectively. For details, see Text S1.

### Statistical analyses.

Unless otherwise specified in the figure legends, all reported results are means ± standard errors (SEs) and have been tested for statistical significance using the permutational *t* test.
