# Supplementary material for: AupA and AupB Are Outer and Inner Membrane Proteins Involved in Alkane Uptake in Marinobacter hydrocarbonoclasticus SP17
Source: mBio. 2018 Jun 5;9(3):e00520-18. doi: 10.1128/mBio.00520-18 (PMC5989066; doi:10.1128/mBio.00520-18)
Supplement: FIG S1 [file mbo003183910sf1.pdf]

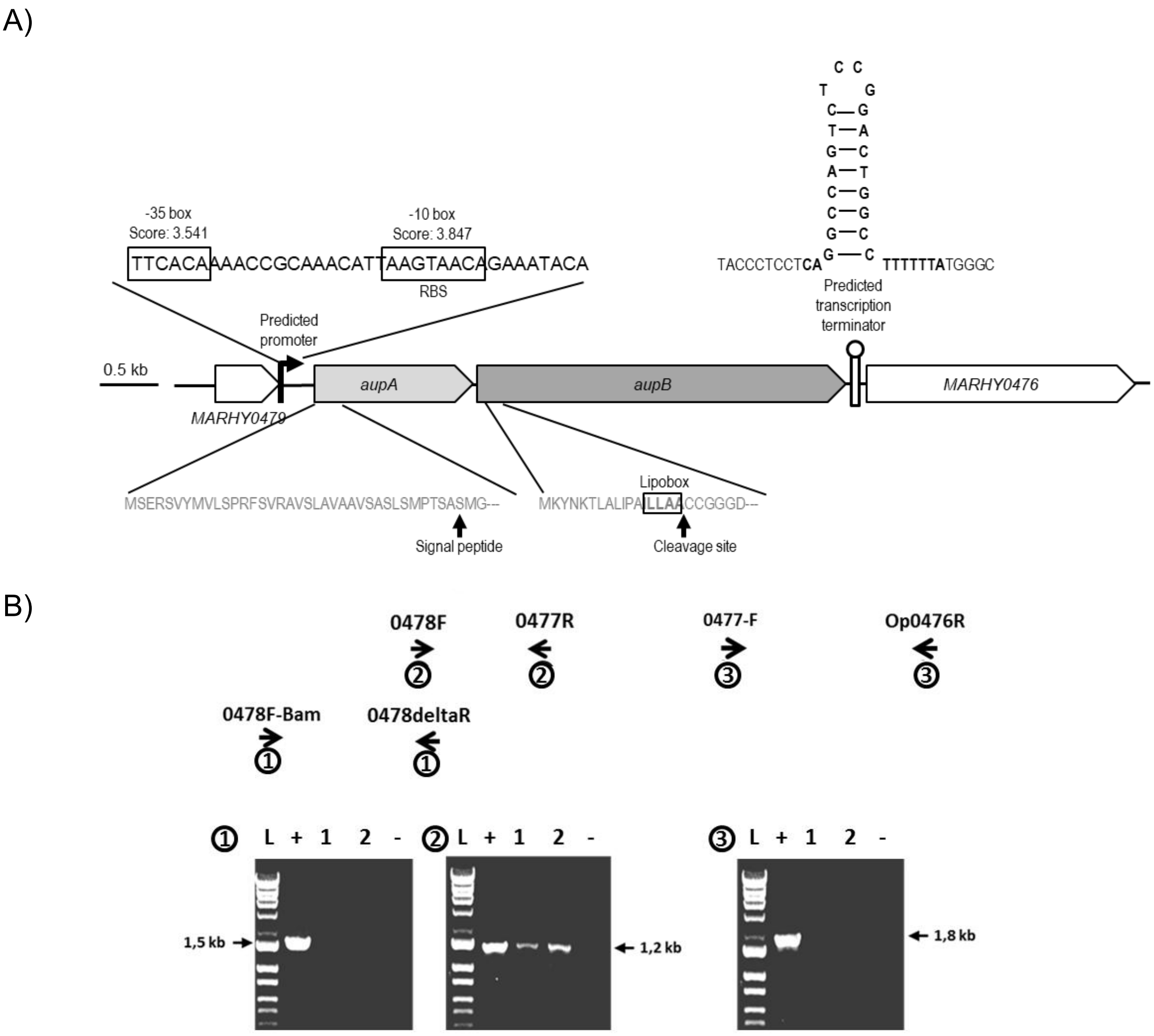

Figure S1: Genomic organization of the *aup* locus.

A) Genetic structure of the *aupAB* operon. Protein sequences are in gray and nucleotide sequences in black. **B) Agarose gel electrophoresis of RT-PCR amplifications of the *aupAB* region.** The positions on the genome of the primers used are indicated. PCR products were obtained with primers 0478F-Bam and 0478deltaR (electrophoresis 1), 0478F and 0477R (electrophoresis 2), 0477-F and Op0476R (electrophoresis 3). DNA templates used were as follows: lane (+), 10 ng of JM1 genomic DNA; lanes 1 and 2, 5 µL of cDNAs obtained from RT reactions on 0.8 µg and 1.6 µg of JM1 total RNAs, respectively; lane (-), 1.6 µg of JM1 total RNAs. Lane L: DNA ladder.

Sigma-70 promoter was predicted using the on line tool, BPROM V. Solovyev, A Salamov (2011) Automatic Annotation of Microbial Genomes and Metagenomic Sequences. In Metagenomics and its Applications in Agriculture, Biomedicine and Environmental Studies (Ed. R.W. Li), Nova Science Publishers, p. 61-78. Transcription terminator was predicted using the ARNOLD web Server (http://rna.igmors.u-psud.fr/toolbox/arnold/index.php). Gautheret D, Lambert A. (2001) Direct RNA Motif Definition and Identification from Multiple Sequence Alignments using Secondary Structure Profiles. J Mol Biol. 313:1003–11. Macke T, Ecker D, Gutell R, Gautheret D, Case DA and Sampath R. (2001) RNAMotif – A new RNA secondary structure definition and discovery algorithm. Nucleic Acids Res. 29:4724–4735. Signal sequence were predicted with SignalP 4.1. SignalP 4.0: discriminating signal peptides from transmembrane regions. Thomas Nordahl Petersen, Søren Brunak, Gunnar von Heijne & Henrik Nielsen Nature Methods, 8:785-786, 2011.
