# Supplementary material for: AupA and AupB Are Outer and Inner Membrane Proteins Involved in Alkane Uptake in Marinobacter hydrocarbonoclasticus SP17
Source: mBio. 2018 Jun 5;9(3):e00520-18. doi: 10.1128/mBio.00520-18 (PMC5989066; doi:10.1128/mBio.00520-18)
Supplement: TEXT S1 [file mbo003183910s1.pdf]

SUPPORTING MATERIALS AND METHODS

All chemicals were purchased from SIGMA-ALDRICH unless otherwise specified.

Culture conditions

M. hydrocarbonoclasticus strains were routinely grown at 30 °C under shaking at 200 rpm in SSW (Synthetic Sea Water) (1) or in Halo medium (0.17 M NaCl, 11.6 mM Na_3-citrate, 81.6 mM $MgSO_4$, 3.4 mM K_2HPO_4, 0.17 mM $Fe(NH_4)_2(SO_4)_2$, 5 g/L proteose peptone, 1 g/L yeast extract). Media were supplemented with 20 mM Na acetate or Na DL-lactate, and 100 µg/mL streptomycin. To cultivate mutant strains, kanamycin was also added at a concentration of 100 µg/mL and for strains harboring a mini-Tn*7*T, gentamycin was used at 30 µg/mL. The growth of JM1 and its derivatives was assessed on various substrates in SSW supplemented with streptomycin. The cultures were performed in triplicate in glass tubes and substrates were directly added to the medium to a final concentration of 10 mM - caproic acid, Tween® 20, Na butyrate, propanoic acid, Na fumarate, succinic acid, Na citrate, Na adipate, Na benzoate, L-proline. The casamino acid was used at 1 g/L and the *n*-propanol at 13 mM. The 3-eicosanone was first solubilized in acetone and put into a glass flask, the solvent was then left to evaporate prior to inoculum addition; its final concentration was 4 mM.

E. coli strains were grown at 37 °C with shaking at 200 rpm in LB (2) or Halo media, supplemented with the appropriate antibiotic when necessary at a concentration of 100 µg/mL unless otherwise specified. Agar was added to a final concentration of 1.4 % for all solid media.

DNA and RNA techniques: materials and instructions

Genomic DNAs were extracted with the Ultraclean Microbial DNA Isolation kit (MoBio) as

described in the user's guide or according to Sambrook and Russell (2) if larger amounts were required. Plasmid DNAs were obtained by mini- or midi-preparations made from 1.5 mL of *E. coli* cell suspensions as described by Sambrook and Russell (2) or by extraction with the "Qiaprep® Spin Miniprep" kit (QIAGEN).

Restriction endonucleases were purchased from Takara Biochemicals or New England Biolabs and T4 DNA ligase was acquired from Promega. DNA fragments were purified using the "GFX PCR DNA and Gel Band Purification" kit (GE Healthcare).

Total RNAs were extracted with EXTRACT-ALL® (EUROBIO) according to the manufacturer's instructions and stored at -80 °C until use. Total RNA intended for RT-qPCR were quantified with the "Quant-it RiboGreen® RNA assay" kit (Invitrogen) and residual DNA was removed from RNA preparations with the "TURBO DNA-free™ kit" (Applied Biosystems) according to the user's guide. RNAs (1.6 µg) were then retro-transcribed in duplicate with the "Superscipt™ III First-Strand Synthesis System for RT-PCR" (Invitrogen) with the supplied random hexamers and according to the manufacturer's instructions. Total RNA destined for RT-PCR were quantified with the "Quant-iT™ RNA Assay Kit" on the fluorometer Qubit® (Invitrogen). DNA traces were removed as described above after a first purification with "RNeasy mini Kit" (QIAGEN). RNAs (0.8 µg or 1.6 µg) were retro-transcribed in cDNAs as described above.

PCR and qPCR

10 ng of genomic DNA, unless otherwise specified, or 5 µL of the RT reactions were used in PCR with 2.5 U of *Taq* DNA polymerase (Eurobio) or 1 U of "i-Proof High Fidelity" (Bio-Rad), their 1X buffer, 0.2 µM of each customized primer synthesized by Eurogentec (Table S3), 0.5 mM

$MgCl_2$ and 0.2 mM dNTPs (Eurobio). PCR products were purified using the "GFX PCR DNA and Gel Band Purification" kit (GE Healthcare) according to the user's guide. The PCRs were carried out on Veriti, GeneAmp PCR System 9700 or 2720 Thermal Cycler (Applied Biosystems).

For qPCR, the RT reaction was diluted three-fold, 5 µL of cDNA were added to the qPCR mix (Applied Biosystems) containing the appropriate primers (Table S3) and the amplification was performed on a Mx3005P thermocycler (Stratagene). Quality of amplifications was assessed by the dissociation curve method and its efficiency was tested with the LinRegPCR software (3). Relative changes in gene expression were calculated with the $2^{-\Delta\Delta Ct}$ method (4) using the 16S rDNA gene as reference gene.

Construction of pKOMKm suicide vector

pKOMKm is derived from the suicide vector pKAS32 (5). The *E. coli rpsL* gene of pKAS32 was replaced by the *M. hydrocarbonoclasticus rpsL* gene amplified from the original *M. hydrocarbonoclasticus* SP17 Sm^S strain with the *0577F_Sal* and *0577R_Sal* primers and inserted into the *Sal*I restriction sites after *Sal*I restriction. A *Xba*I fragment carrying the kanamycin resistance cassette from pMKm (6) was inserted into the *Xba*I site of the pKAS32 multicloning site (MCS). The resulting plasmid allows the cloning of gene fragments on both sides of the kanamycin cassette to create knockout alleles. The *M. hydrocarbonoclasticus* wild-type *rpsL* gene can complement the *rpsLK58T* allele conferring resistance to streptomycin in JM1, providing antibiotic selection for allelic exchange in *M. hydrocarbonoclasticus.*

Construction of knockout mutants

To construct JM2 (Δ*aupA*), a 838-bp long fragment and a 628-bp long fragment overlapping

the 5’ and 3’ ends and their flanking regions of the *aupA* gene were amplified using the *0478F-Bam/Bgl0478-2* and *Bgl0478F/0478R-Not* primer pairs, respectively, and cloned into pCR™2.1-TOPO® (Table S3). A truncated version of the *aupA* gene, which contained a central unique *Bgl*II restriction site, was obtained by ligating both fragments together after their restriction with *Bam*H1 and *Bgl*II or *Bam*HI and *Not*I, respectively. and their cloning into the *Bam*HI-*Not*I double digested pKAS32 vector. This vector was used according to the strategy described by Ortiz-Martin *et al.* (7) to generate pKAS32-Δ*aupA*::*aphA*, a suicide plasmid carrying a *Bgl*II-digested kanamycin cassette in the *Bgl*II site of the truncated *aupA* gene. The orientation of the cassette was confirmed by sequencing. JM3 (Δ*aupB*) and JM4 (Δ*aupAB*) mutants were constructed using the pKOMKm suicide vector. A 660-bp long fragment and a 656-bp long fragment overlapping the 5’ and 3’ ends and their flanking regions of the *aupA* gene were amplified using the *0477F-EcoRV/0477R-NheI* and *0477F-KpnI/0477R-NdeI* primer pairs, respectively. They were inserted into pKOMKm to generate the pKOMKm-Δ*aupB*::*aphA* plasmid. A 1079-bp long fragment and a 1171-bp long fragment overlapping the 5’ and 3’ ends and their flanking regions of the *aupA-aupB* operon were amplified using the *0478pKOM-EcoRI/0478pKOM-KpnI* and *0477pKOM-NheI/0477pKOM-EcoRV* primer pairs, respectively. They were inserted into pKOMKm to generate the pKOMKm-Δ*aupAB*::*aphA* plasmid. Suicide plasmids were introduced into JM1 (WT) by one-parental mating conjugation using *E. coli* S17-1 (λpir) as a donor. The conjugation was realized at 30 °C during 24 h on cellulose acetate filters (0.45 µm pore size; Millipore) using the Halo medium supplemented with lactate. Transconjugants were selected on solid SSW containing lactate and kanamycin. The resulting colonies were replicated on solid SSW containing ampicillin (200 µg/mL) or streptomycin to determine whether each trans-conjugant was the result of plasmid integration or allelic exchange. Candidate mutants ($Km^R/Amp^S/Sm^R$) were checked by PCR for

the insertion of the mutant allele at the desired locus using the following primer pairs: *aphaF-A*/*0479-F* and *aphaR-A*/*Mh0478R* for JM2, *aphaF-A*/*0478F* and *aphaR-A*/*Op0476R* for JM3, *aphaF-A*/*0476jonction-F* and *aphaR-A*/*0480jonction-R* for JM4 (Table S3). The absence of the wild-type allele was tested with *0478delta-F*/*0478delta-R*, *Mh0477F*/*Mh0477R* or *0478F*/*0477R* primer pairs in JM2, JM3 or JM4, respectively. The presence of the kanamycin cassette or of the *bla* gene carried by the suicide plasmid was tested with *Km aphA-F*/*Km aphA-R* and *pKAS32amp-F*/*pKAS32amp-R* primer pairs, respectively.

Construction of complemented strains using pUC18T-mini-Tn*7*T-Gm derivatives

The *aupA* and *aupB* genes and the *aupAB* operon were cloned into pUC18T-mini-Tn*7*T-Gm vectors (8). The *aupA* gene and its promoter sequence was amplified with the *0478F-Spe*/*0478R-Hind* primer pair. The *aupA-aupB* operon was obtained by PCR that amplified three fragments overlapping the full operon and its promoter sequence with the *Mh0478F*/*Mh0477R*, *0478F-Spe*/*RT0478* and *Mh-0477F*/*0477R-Nru* primer pairs, respectively. The 5' fragment was digested with *Spe*I and *Bam*HI, the central fragment was digested with *Bam*HI and *Hind*III, and the 3' fragment was digested with *Hind*III and *Sfi*I. The whole operon was then obtained as a *Spe*I-*Sfi*I fragment after ligation. These two genes were then under the control of the native promoter of the *aupA-aupB* operon. The *aupB* gene was obtained by ligating two restricted-PCR fragments amplified with *0477F-NruI*/*Mh_477R* and *0477-F*/*0477R-StuI* primer pairs. Its expression was obtained by transcriptional fusion with the $P_{A1/04/03}$ promoter amplified from the pUC18T-mini-Tn*7*T-Gm-*eyfp* vector with the *PA1/04/03F-ApaI/PA1/04/03R-NruI* primer pair. All the constructs were verified by sequencing. Insertion of mini-Tn*7*T derivatives in the genomes of the *M. hydrocarbonoclasticus* strains was performed by four-parental mating conjugation as described

by Choi *et al*. (8), with modifications described above. Basically, the mini-Tn*7*T delivery was *E. coli* DH5α/pUC18T-mini-Tn*7*T-Gm derivatives and the helper strains were SM10(λpir)/pTNS2 and HB101/pRK2013. The selection of trans-conjugants was realized on SSW plates containing lactate, streptomycin and gentamycin (30 µg/mL). Insertion at the *M. hydrocarbonoclasticus attTn7* site was checked by PCR with the *marhy3826rev*/*pTn7L* and *pmarhyglmS*/*pTn7R* primer pairs. The presence of the gentamycin resistance gene carried by the mini-Tn*7*T was PCR-verified with the *Gm-up*/*Gm-down* primer pair.

Confocal Laser Scanning Microscopy and image analysis

Cells were observed using a Leica SP2 AOBS inverted confocal laser scanning microscope (LEICA Microsystems, France) at the MIMA2 microscopy platform (http://www6.jouy.inra.fr/mima2). For observations of bacteria on paraffin, cells were grown on 1-mm^2 glass slides coated with a thin layer of paraffin. Slides were turned upside down and deposited on a glass coverslip (0.15-mm thick) with a silicone spacer (1-mm thick) and bacteria were stained with 5 µM Syto 9 (Invitrogen, France). After 15 min of incubation in the dark at room temperature, the samples were scanned at 400 Hz with a water immersion objective 40X at numerical zoom 4 for paraffin assays and with an oil immersion objective 63X numerical zoom 4 for *n*-hexadecane assays. Syto 9 excitation was performed at 488 nm with an argon laser, and the emitted fluorescence was recorded within the range 500 nm–600 nm. Four stacks of horizontal plane images with a z-step of 1 µm were acquired for each sample. Easy 3D blend projections of surface associated bacteria on paraffin were reconstructed from Z-series images using IMARIS v7.2 software (Bitplane, Switzerland). The biovolume, subtratum coverage, mean thickness and roughness of biofilms were calculated using the PHLIP Matlab routine. The quantification of

bacterial adhesion to *n*-hexadecane was performed with ImageTool 3.0 software (http://imagetool.software.informer.com/3.0/).

Bioinformatics analyses

Predictions of promoter and transcription terminator positions on the *aupAB* operon were performed using the PePPER (9) and ARNold (10) software, respectively. The presence of signal peptides in AupA and AupB proteins was evaluated using the SignalIP-4.1 web tool (11). The search for *aupA* and *aupB* homologs were performed in GenBank using the BLAST program (12) and genetic organization was examined with ARTEMIS (13).

Protein purification and antisera preparation

The ORF of *aupA* was amplified with the *TopoF0478*/*TopoR0478His* primer pair and the product was cloned into the pBAD-TOPO® vector using the "pBAD-TOPO® TA Expression Kit" (Invitrogen) and the host strain LMG194 to give the pBAD-AupAHis. Cells of LMG194/pBAD-AupAHis were grown in LB at 37 °C to an $OD_{600\ nm}$ of 0.7, 0.2 % (v/v) arabinose were added and growth was continued for 3 h at 37 °C and 200 rpm. Cells were harvested by centrifugation at 12,000 x g for 30 min at 4 °C and 1.85 g of wet cells were resuspended in 6.5 mL of buffer A (Hepes 25 mM pH 7.5, NaCl 300 mM, glycerol 10 % (v/v)). Cells were lysed by a single passage through an ice-chilled French press at 40,000 psi. Insoluble proteins were collected by centrifugation at 40,000 rpm for 40 min at 4 °C and resuspended in 2 mL of buffer A supplemented with 1 % (v/v) N,N-diméthyldodécylamine N-oxyde (DDAO). After centrifugation at 20,000 x g for 30 min at 4 °C, the supernatant (2 mL) was applied to a 1 mL Ni^{2+}-sepharose column (Bio-Rad), washed with 10 volumes of buffer B (buffer A supplemented with 0.2 % (v/v) DDAO and

10 mM imidazole) and AupAHis was eluted by a 5 mL imidazole gradient from 0 to 250 mM. The AupAHis containing fractions were pooled, concentrated by ultrafiltration on a 3 kDa cut off Amicon UItra filter (Millipore) and applied to a 1.5 mL Superdex 200 HR 10/30 column (GE Healthcare). The column was run with buffer C (Hepes 25 mM pH 7.5, NaCl 100 mM, glycerol 10 % (v/v), DDAO 0.05 % (v/v)) and fraction containing AupAHis was stored at -80 °C.

The ORF of *aupB* was amplified using the *pb0477pET-Nde* and *pb0477pET-Not* primers and was cloned into *Nde*I and *Not*I sites of the pET-21a(+) using the sites engineered onto the primers to give pET-AupBHis. The strain BL21 (DE3) was used as host for expression of AupBHis. Cells of BL21 (DE3)/pET-AupBHis were grown in LB at 37 °C to an $OD_{600\ nm}$ of 0.7, 1 mM isopropyl β-D-galactopyranoside (IPTG) was added and the culture was incubated for 3 h at 37 °C and 200 rpm. Cells were harvested by centrifugation at 12,000 x g for 30 min at 4 °C and 6.5 g of wet cells were resuspended in 13 mL of buffer A2 (Hepes 25 mM pH 7.5, NaCl 200 mM). The cells were lysed by a single passage through an ice-chilled French press at 40,000 psi (Thermo Spectronic). AupBHis essentially produced as inclusion bodies was collected by centrifugation at 15,000 x g for 30 min at 4 °C and resuspended in 13 mL of buffer B2 (buffer A2 supplemented with 8 M urea). After centrifugation at 15,000 x g for 40 min at 4 °C, the supernatant (10 mL) was applied to a 2 mL Immobilized Metal Affinity Chromatography (IMAC) column (Bio-Rad), washed with 10 volumes of buffer B2 and the recombinant AupBHis was eluted with a 35 mL linear gradient of imidazole from 0 to 500 mM. The fractions containing AupBHis (6 mL) were then applied to a 2 mL anion exchange Q sepharose column (GE Healthcare), washed with 15 volumes of buffer C2 (25 mM Tris-HCl pH 7.5, 8 M urea) and the protein was eluted with a 30 mL linear gradient

The production of rabbit polyclonal antibodies raised against the C-terminal His-tagged

version of AupA and AupB was subcontracted to the Proteogenix company (http://www.proteogenix-antibody.com/fr/).

SDS-PAGE and immunoblotting

Proteins from 2 ml of planktonic cultures at $OD_{600\ nn}$=0.3-0.5 or from biofilm cell suspensions were precipitated with 5 % trichloroacetic acid (TCA) for 1 h at 4 °C, pelleted by centrifugation for 5 min at 20,000 x g at 4 °C and washed twice with 1 mL of cold acetone. Protein pellets were air-dried and dissolved in SDS-PAGE sample buffer (2). Protein concentration was estimated using the "QuantiPro™ BCA assay kit" (SIGMA-ALDRICH) according to the manufacturer's instructions. For each extract, 25 µg of total proteins were separated by SDS-PAGE 10 % (w/v) and electro-transferred onto PVDF membranes (Hybond-P, Amersham Pharmacia Biotech) at 50 V for 1 h in a buffer consisting of 25 mM Tris-HCl pH 8.3, 192 mM glycine and 10 % (v/v) ethanol. The membranes were saturated for 30 min in PBS buffer (154 mM NaCl, 0.2 mM KH_2PO_4, pH 7.4) containing 0.1 % (v/v) of Tween® 20 and 5 % (w/v) of skim milk. Primary antibody was then added with a dilution of 1/5,000 for anti-AupA antiserum and of 1/10,000 for anti-AupB antiserum, and incubation was continued overnight at room temperature. The detection was performed with a secondary antibody linked to the horseradish peroxidase and the "ECL™ western blotting detection reagents" (GE Healthcare) and imaging was performed with Typhoon 9200 scanner (GE Healthcare) as indicated by the manufacturer.

Immunoprecipitation assays

Immunoprecipitation assays were carried out on cell lysates prepared from 5.10^{11} cells in exponential growth phase ($OD_{600\ nm}$~0.5) on SSW-acetate that were centrifuged at 10,000 x g for

15 min at 4 °C, washed twice with 150 mL of PBS (137 mM NaCl, 2.7 mM KCl, 10 mM Na_2HPO_4 and 2 mM KH_2PO_4) and resuspended in 10 mL of cold lysis buffer (150 mM NaCl, 50 mM Tris-HCl pH 8, 1 % (v/v) Triton™ X-100, 0.5 % (v/v) sodium deoxycholate, 0.1 % (v/v) SDS and 8.6 mg/mL protease inhibitor cocktail). The cells were then lysed by sonication for 1 min at 35 W and 500 ms/s pulses (Sonifier 450, Branson) and the lysates (supernatant) were recovered by centrifugation at 5,000 x g for 5 min at 4 °C. 400 µL of anti-AupA antiserum was added to 1.6 mL of the lysates freshly prepared and the reaction was incubated at 4 °C for 4 h under agitation. 200 µL of protein-A-sepharose beads (prepared in 10 % (v/v) of lysis buffer without protease inhibitor cocktail) were then added to the immune complex and the incubation was continued for 1 h in the same conditions. The beads were collected by centrifugation at 10,000 x g for 20 s at 4 °C, washed three times with lysis buffer (without protease inhibitor cocktail). The liquids were removed by aspiration with a 23-gauge needle bent linked to an aspiration system. For the final wash, the needle was inserted directly into the beads to remove the remaining wash buffer. The presence of AupB was detected by immunoblotting as previously described.

NADH oxidase activity and KDOs assays

NADH oxidase activity was measured with 20 µL of each fraction diluted in 180 µL of Tris-HCl 50 mM pH 7.5, dithiothreitol 0.2 mM and NADH 0.5 mM. Absorbance at 340 nm was recorded for 15 min at 25 °C using double beam spectrophotometer (UVIKON XL) and the values were converted into concentration of NAD+ produced using ε_{NADH}=6,220 $M^{-1}.cm^{-1}$. KDOs assays were performed on 180 µL of each fraction according to the method described by Osborn *et al.* (14).

Hexadecane degradation assays

Hexadecane degradation assays were carried out in stoppered 50 mL glass serum bottles containing 5 mL of SSW medium with 1,095 µg/mL *n*-hexadecane. Cultures were inoculated at an $OD_{600\ nm}$ of 0.1. After incubation, cultures were extracted with 2 mL of methanol and 5 mL of dichloromethane containing 100 µg/mL dodecane as an internal standard and sonicated for 8 min in an ultrasound bath (SONOREX, Bandelin). Analysis and quantification of extracted alkanes were performed on a GC system 6850 Network (Agilent Technologies) equipped with a 190912-413E capillary column (Agilent; 30 m long; 320 µm diameter; 0.25 µm film thickness) and a FID detector (air/hydrogen with 350 mL/min and 35 mL/min outputs respectively). The oven temperature was programmed to rise from 60 °C to 172 °C with three different rates: 20 °C/min up to 130 °C, 1.5 °C/min up to 150 °C and 4 C/min up to 172 °C. The carrier gas was helium at 0.4 bar. All degradation experiments were performed in triplicate.

SUPPLEMENTAL REFERENCES

1. **Gauthier MJ, Lafay B, Christen R, Fernandez L, Acquaviva M, Bonin P, et al.** 1992. *Marinobacter hydrocarbonoclasticus* gen. nov., sp. nov., a new, extremely halotolerant, hydrocarbon-degrading marine bacterium. Int J Syst Bacteriol. **42**:568–76.
2. **Sambrook JJ, Russell DDW.** 2001. Molecular cloning: a laboratory manual. Vol. 2. CSHL Press.
3. **Ramakers C, Ruijter JM, Deprez RHL, Moorman AFM.** 2003. Assumption-free analysis of quantitative real-time polymerase chain reaction (PCR) data. Neurosci Lett. **339**(1):62-66.
4. **Livak KJ, Schmittgen TD.** 2001. Analysis of Relative Gene Expression Data Using Real-Time Quantitative PCR and the $2^{-\Delta\Delta CT}$ Method. Methods. **25**(4):402-408.

5. **Skorupski K, Taylor RK.** 1996. Positive selection vectors for allelic exchange. Gene. **169**(1):47-52.

6. **Murillo J, Shen, H., Gerhold, D., Sharma, A., Cooksey, D. A. and Keen, N. T.** 1994. Characterization of pPT23B, the plasmid involved in syringolide production by *Pseudomonas syringae* pv. tomato PT23. Plasmid. **31**(3):275-287.

7. **Ortiz-Martín I, Macho AP, Lambersten L, Ramos C, Beuzón CR.** 2006. Suicide vectors for antibiotic marker exchange and rapid generation of multiple knockout mutants by allelic exchange in Gram-negative bacteria. J Microbiol Methods. **67**(3):395-407.

8. **Choi, K.H., Gaynor, J.B., White, K.G., Lopez, C., Bosio, C.M., Karkhoff-Schweizer, R.R. and Schweizer, H.P.** 2005. A Tn7-based broad-range bacterial cloning and expression system. Nat Methods. **2**:443-448.

9. **De Jong A, Pietersma H, Cordes M, Kuipers OP, Kok J.** 2012. PePPER: a webserver for prediction of prokaryote promoter elements and regulons. Bmc Genomics. **13**(2):299.

10. **Naville M, Ghuillot-Gaudeffroy A, Marchais A, Gautheret D.** 2011. ARNold A web tool for the prediction of Rho-independent transcription terminators. Rna Biol. **8**(1):11–3.

11. **Petersen TN, Brunak S, von Heijne G, Nielsen H.** 2011. SignalP 4.0: discriminating signal peptides from transmembrane regions. Nat Methods. **8**(10):785–6.

12. **Altschul SF, Gish W, Miller W, Myers EW, Lipman DJ.** 1990. Basic local alignment search tool. J Mol Biol. **215**(3):403–10.

13. **Rutherford K, Parkhill J, Crook J, Horsnell T, Rice P, Rajandream M-A, et al.** 2000. Artemis: sequence visualization and annotation. Bioinformatics. **16**(10):944–5.

14. **Osborn MJ, Gander JE, Parisi E.** 1972. Mechanism of assembly of the outer membrane of *Salmonella typhimurium*. J Biol Chem. **247**(12):3973-86.
