# Supplementary material for: AupA and AupB Are Outer and Inner Membrane Proteins Involved in Alkane Uptake in Marinobacter hydrocarbonoclasticus SP17
Source: mBio. 2018 Jun 5;9(3):e00520-18. doi: 10.1128/mBio.00520-18 (PMC5989066; doi:10.1128/mBio.00520-18)
Supplement: FIG S2 [file mbo003183910sf2.pdf]

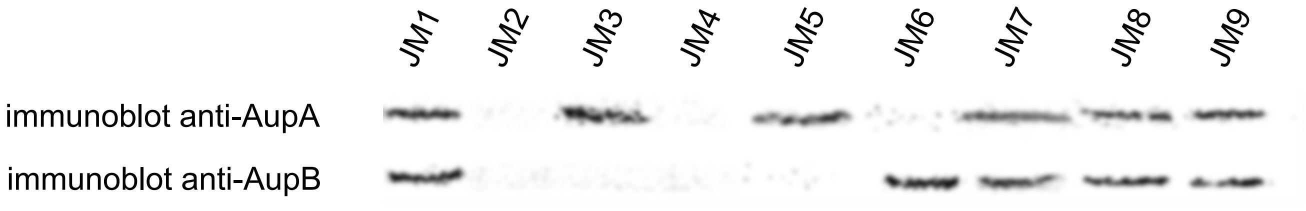

Figure S2: Immunoblots on Δ*aup* mutants and complemented strains.
Total protein extracts from *M. hydrocarbonoclasticus* strains grown on SSW-acetate were analyzed by western-blot using either anti-AupA or anti-AupB antiserum. JM1, wild-type strain; JM2, Δ*aupA::aphA* knockout mutant in JM1; JM3, Δ*aupB::aphA* knockout mutant in JM1; JM4, Δ*aupAB::aphA* knockout mutant in JM1; JM5, JM2 that carries wild-type *aupA* under its own promoter on a mini-Tn7T; JM6, JM2 that carries wild-type *aupB* under $PA_{1/04/03}$ promoter on a mini-Tn7T; JM7, JM2 that carries wild-type *aupAB* under its own promoter on a mini-Tn7T; JM8, JM3 that carries wild-type *aupB* under $PA_{1/04/03}$ promoter on a mini-Tn7T; JM9, JM4 that carries wild-type *aupAB* under its own promoter on a mini-Tn7T.
