# Supplementary material for: AupA and AupB Are Outer and Inner Membrane Proteins Involved in Alkane Uptake in Marinobacter hydrocarbonoclasticus SP17
Source: mBio. 2018 Jun 5;9(3):e00520-18. doi: 10.1128/mBio.00520-18 (PMC5989066; doi:10.1128/mBio.00520-18)
Supplement: TABLE S1 [file mbo003183910st1.pdf]

Table S1: Bacterial strains, plasmids and primers.

Marinobacter hydrocarbonoclasticus strains

train number	Relevant genotype/phenotype	Characteristics	Source
SP17<br>ATCC 49840	Wild-type	Original isolate	(1)
JM1	SP17, *rpsL*K58T; Spontaneous streptomycin resistant mutant	Referred to as wild-type (WT)	This study
JM1/Tn7	JM1, *att*Tn*7*::[mini-Tn*7*T-Gm]; Sm^R, Gm^R	JM1 strain containing a mini-Tn*7*T	This study
JM2	JM1, Δ*aupA::aphA ;* Sm^R, Km^R	*aupA* knockout mutant in JM1	This study
JM3	JM1, Δ*aupB::aphA ;* Sm^R, Km^R	*aupB* knockout mutant in JM1	This study
JM4	JM1, Δ*aupAB::aphA ;* Sm^R, Km^R	*aupAB* knockout mutant in JM1	This study
JM5	JM1, Δ*aupA::aphA, att*Tn*7*::[mini-Tn*7*T-Gm-*aupA*]; Sm^R, Km^R, Gm^R	Carries wild-type *aupA* under its own promoter on a mini-Tn*7*T	This study
JM6	JM1, Δ*aupA::aphA, att*Tn*7*::[mini-Tn*7*T-Gm-$P_{A1/04/03}$-*aupB*]; Sm^R, Km^R, Gm^R	Carries wild-type *aupB* under $P_{A1/04/03}$ promoter on a mini-Tn*7*T	This study
JM7	JM1, Δ*aupA::aphA, att*Tn*7*::[mini-Tn*7*T-Gm-*aupAB*]; Sm^R, Km^R, Gm^R	Carries wild-type *aupAB* under its own promoter on a mini-Tn*7*T. Used for complementation	This study
JM8	JM1, Δ*aupB::aphA, att*Tn*7*::[mini-Tn*7*T-Gm-$P_{A1/04/03}$-*aupB*] ; Sm^R, Km^R, Gm^R	Carries wild-type *aupB* under $P_{A1/04/03}$ promoter on a mini-Tn*7*T. Used for complementation	This study
JM9	JM1, Δ*aupAB::aphA, att*Tn*7*::[mini-Tn*7*T-Gm-*aupAB*]; Sm^R, Km^R, Gm^R	Carries wild-type *aupAB* under its own promoter on a mini-Tn*7*T. Used for complementation	This study

Escherichia coli strains

train number	Relevant genotype/phenotype	Characteristics	Source
JM109	*rec*A1*, end*A1*, gyr*A96*, thi, hsd*R17 (r_K–,m_K+), *rel*A1*, sup*E44, Δ*(lac-pro*AB*)*, [F´, *tra*D36*, pro*AB*, lacI*^{q}ZΔM15]	Host for cloning	Promega
TOP10F’	F’ {*lac*I^q *Tn*10 (Tet^R)} *mcr*A Δ*(mrr-hsd*RMS-*mcr*BC*)* Φ80*lac*ZΔM15 Δ*lac*X74 *rec*A1 *ara*D139 Δ(*ara-leu*)7697 *gal*U *gal*K *rpsL endA1 nupG;* Sm^R	Host for cloning	Invitrogen
LMG194	F- Δ*lac*X74 *gal*E *thi rps*L Δ*pho*A *(Pvu* II*)* Δ*ara*714 *leu::Tn*10	Host for protein expression	Invitrogen
BL21 (DE3)	F- *omp*T *gal dcm lon hsd*SB(r_B- m_B-) λ(DE3 [*lac*I *lac*UV5-T7 gene 1 *ind*1 *sam*7 *nin*5])	Host for protein expression	(2)
S17-1 (λpir)	*rec*A, *thi, pro, hsd*R-M+RP4: 2-Tc:Mu:Km Tn*7* λpir; Sm^R Tp^R	Donor strain in two-parental mating conjugation	(3)
DH5α	F- *end*A1 *gln*V44 *thi*-1 *rec*A1 *rel*A1 *gyr*A96 *deo*R *nup*G Φ80d*lac*ZΔM15 Δ(*lacZYA-argF)*U169*, hsd*R17(r_K- m_K+), λ-	Host for cloning and donor strain in four-parental mating conjugation	(4)
SM10 (λpir) / pTNS2	*thi*-1*, thr, leu, ton*A*, lac*Y*, sup*E*, rec*A::RP4-2-Tc::Mu*, pir* / pTNS2 (Amp^R *ori*R6K, *tnsABCDE*); Km^R	Helper strain in four-parental mating conjugation	(5)

HB101 / pRK2013	F- *mcr*B *mrr hsd*S20(rB- mB-) *rec*A13 *leu*B6 *ara*-14 *pro*A2 *lac*Y1 *gal*K2 *xyl*-5 *mtl*-1 *rpsL*20 (Sm^R) *gln*V44 λ- / pRK2013 (Km^R *ori*ColE1 RK2-Mob+ RK2-Tra+)	Helper strain in four-parental mating conjugation	(5)

Plasmids

**Name**	**Relevant genotype**	**Characteristics**	**Source**
pBAD-TOPO®	Amp^R	cloning and expression vector	Invitrogen
pBAD-AupAHis	pBAD-TOPO® carrying *aupA* CDS with 6 His codon in 3' end, Amp^R, Km^R	used for AupA expression	This study
pCR^{TM}2.1-TOPO®	Amp^R	cloning vector	Invitrogen
pET-21a(+)	pET-21a(+) carrying *aupB* CDS with 6 His codon in 3' end	cloning and expression vector	Novagen
pET-AupBHis	Amp^R	used for AupB expression	This study
pGEM®-T	Amp^R	cloning vector	Promega
pKAS32		cloning and suicide vector	(6)
pKAS32-*aupA*	pKAS32 carrying *aupA* and flanking regions		This study
pKAS32-*ΔaupA*::*aphA*	pKAS32 carrying Δ*aupA::aphA*	used for knocking-out *aupA*	This study
pKOMKm	Amp^R, Km^R	cloning and suicide vector	This study
pKOMKm-*ΔaupB*::*aphA*	pKOMKm carrying Δ*aupB::aphA*	used for knocking-out *aupB*	This study
pKOMKm-*ΔaupAB::aphA*	pKOMKm carrying Δ*aupAB::aphA*	used for knocking-out *aupAB*	This study
pUC18T-mini-Tn7T-Gm	Gm^R	cloning vector	(7)
pUC18T-mini-Tn7T-Gm-*eyfp*	pUC18T-mini-Tn7T-Gm carrying *eyfp* gene	used to take $P_{A1/04/03}$ promoter	(5)
pUC18T-mini-Tn7T-Gm-*aupA*	pUC18T-mini-Tn7T-Gm carrying *aupA* gene cloned with its own promoter	used for Δ*aupA::aphA* complementation	This study
pUC18T-mini-Tn7T-Gm-$P_{A1/04/03}$-*aupB*	pUC18T-mini-Tn7T-Gm carrying *aupB* gene cloned with the $P_{A1/04/03}$ promoter from pUC18T-mini-Tn7T-Gm-*eyfp*	used for Δ*aupB* complementation in Δ*aupAB::aphA* mutant	This study
pUC18T-mini-Tn7T-Gm-*aupAB*	pUC18T-mini-Tn7T-Gm carrying *aupAB* operon cloned with its own promoter	used for Δ*aupAB::aphA* complementation	This study

Primer pairs		
**Names**	**Sequence[(b)]**	**Source**
0478delta-F/0478delta-R	CGGGCGGTATCCCTTGCCG / GCCCAGATCGATGTTCGTG	This study
0478F/0478R	CCATCGACTCATTCCAGCC / TCACCGTCGTAGTCCACCA	This study
0478F-Bam/0478R-Not	ATGCCAGTGGATCCTAACACACGTTATTGCGGCTCTGG / CATGATGGCGGCCGCTACGTGGATATCACCATCGGCAGT	This study
0478F-Spe/0478R-Hind	ATGTTAGTACTAGTGACGCCGACAAAACTGTCGA / AGCATGATGAAGCTTTCAGAACTTCAGGGTGATGG	This study
0478FpKOM-EcoRI/0478RpKOM-KpnI	ATGACGCGAATTCCTACTCAAGGCTCTCATAATCCC / AGCATGATGGGTACCAGGGATTCCTTTCAGGACTGG	This study
TopoF0478/TopoR0478his	TGAATCTGCCGTGAGGAATAATAAATGAGTGAACGGAGT / TCAGAACTTCAGGGTGATGGA	This study
0478F-Spe/RT0478	ATGTTAGTACTAGTGACGCCGACAAAACTGTCGA / ATGGCAAGCCTCCTCAGAAC	This study
Bgl0478F/Bgl0478-2	ATGCCAGTAGATCTATTTTGTCCTGAATCTGACTGTTT / ATGCCAGTAGATCTCTCCGTTCACTCATTGTTTTT	This study
0477FpKOM-NheI/0477pKOM-EcoRV	ATGACGCGCTAGCTTGGGGCCACAATCACAATC / AGCATGATGGATATCCCGGCTGTGGCGCTTACC	This study
0477F/0477R	TCGATGGTCTATTCCTTCC / CGTAATGCGGTTGCCTTT	This study
pb0477pET-Nde/ pb0477pET-Not	ATGCTAGCCATATGTGCGGTGGTGGCGACAAAC / GTATGCATGCGGCCGCTTCGTACTGAGCCGGAATTT	This study
Mh0477F/Mh0477R	GAGACTTGGAAAACACGACC / CAAAGGCAACCGCATTACG	This study
0477F-EcoRV/0477R-NheI	ATGCCAGGATATCGAGGCTTGCCATGAAGT / AGCATGTGCTAGCGTTGCCTTTGACCAGTA	This study
0477F-KpnI/0477R-NdeI	ATGCCAGGGTACCGACGGTGCCGATTGCC / AGCATGTCATATGGCCTGAGGAGGGTAGACC	This study
0477F-NruI/Mh_477R	ATGCCTCTCGCGAATGAAGTACAACAAGACCC / TCCGTATTCAGAGCGTAGGT	This study
0477-F/0477R-StuI	GTCCTGAACCAACAGGTGGC / AGCATGAAGGCCTCTATTCGTACTGAGCCGGA	This study

Mh-0477F/0477R-NruI	GCATTGCTCTGGTTCGGGA / AGCATGATCGCGACTATTCGTACTGAGCCGGA	This study
Mh0478F/Mh0477R	ACAAGCGTACCAGTCCTGAA / CGTCACCGGCGTTGTTGGCGT	This study
Gm-up/Gm-down	TGGAGCAGCAACGATGTTAC / TGTTAGGTGGCGGTACTTGG	This study
marhy3826rev/pTn7L	CCTGACACCTTGTAACCATTCAG / ATTAGCTTACGACGCTACACCC	This study
pmarhyglmS/pTn7R	AATCTCTTACATCCACGCCG / CACAGCATAACTGGACTGATTTC	This study
pKAS32amp-F/ pKAS32amp-R	GGTCTGACAGTTACCAATGC / GAAGAGTATGAGTATTCAAC	This study
Km aphA-F/Km aphA-R	ATGAGCCATATTCAACGG / AGCATCAAATGAAACTGC	This study
aphaF-A/0478F	CCTCGAGATCTCCATGGAC / CCATCGACTCATTCCAGCC	This study
aphaR-A/Op0476R	CTTGCATGCCTGCAGGTCGA / ATACAGATGAACAGGTCGGTATTG	This study
aphaF-A/0476jonction-F	CCTCGAGATCTCCATGGAC / ATGGTGCTGGCCGGGTACTCT	This study
aphaR-A/0480jonction-R	CTTGCATGCCTGCAGGTCGA / CCGCAACCACAGTTGCTTCACTG	This study
aphaF-A/0479-F	CCTCGAGATCTCCATGGAC / GGGCTTGCCCTGAGAGAAGGTGAAAAATG	This study
aphaR-A/Mh0478R	CTTGCATGCCTGCAGGTCGA / CCGGGTCTGGGTTTTTCGTC	This study
$P_{A1/04/03}$F-ApaI/ $P_{A1/04/03}$R-NruI	ATGCCAGGGGCCCGAAAATTTATCAAAAAGAG / AGCATGTTCGCGATTTCTCCTCTTTAATTCTAG	This study
0577F_Sal/0577R_Sal	ATGCCAGTGTCGACGTGGTGGTAGGGCGTCTGATT / AGCATGATGGTCGACAGCAACGATAAAAGACACCCGC	This study
qMh16SF/qMh16SR	TGAGGCTTGACGTTACCTACA / CTTTCACATCTCGCTTACCAA	This study
qMh477F/qMh477R	GTGGTGGCGACAAACAGT / CAGGGTTGCTTCATCATCA	This study
qMh478F/qMh478R	CAGCACTGACGAAAGATCCT / CTAGGGGAGTCGGAGACAAT	This study

Km^R, Gm^R, Sm^R: resistances to kanamycin, gentamycin, streptomycin.

(a) Amp^R, Km^R, Gm^R, Tet^R, Sm^R, Tp^R: resistances to ampicillin, kanamycin, gentamycin, tetracyclin, streptomycin, trimethoprim.

(b) Restriction sites provided by primers are underlined.

SUPPLEMENTAL REFERENCE

1. **Gauthier MJ, Lafay B, Christen R, Fernandez L, Acquaviva M, Bonin P, et al.** 1992. *Marinobacter hydrocarbonoclasticus* gen. nov., sp. nov., a new, extremely halotolerant, hydrocarbon-degrading marine bacterium. Int J Syst Bacteriol. **42**:568–76.

2. **Studier FW, Moffatt BA.** 1986. Use of bacteriophage T*7* RNA polymerase to direct selective high-level expression of cloned genes. J Mol Biol. **189**(1):113-130.

3. **Simon R, Priefer U, Puhler A.** 1983. A Broad Host Range Mobilization System for Invivo Genetic Engineering Transposon Mutagenesis in Gram-Negative Bacteria. Bio-Technol. **1**(9):784-791.

4. **Hanahan D.** 1983. Studies on Transformation of *Escherichia coli* with Plasmids. J Mol Biol. **166**(4):557-580.

5. **Choi KH, Schweizer HP.** 2006. mini-Tn*7* insertion in bacteria with single attTn*7* sites: Example *Pseudomonas aeruginosa*. Nat Protoc. **1**:153-161.

6. **Skorupski K, Taylor RK.** 1996. Positive selection vectors for allelic exchange. Gene. **169**(1):47-52.

7. **Choi, K.H., Gaynor, J.B., White, K.G., Lopez, C., Bosio, C.M., Karkhoff-Schweizer, R.R., and Schweizer, H.P.** 2005. A Tn*7*-based broad-range bacterial cloning and expression system. Nat Methods. **2**:443-448.
