# Supplementary material for: AupA and AupB Are Outer and Inner Membrane Proteins Involved in Alkane Uptake in Marinobacter hydrocarbonoclasticus SP17
Source: mBio. 2018 Jun 5;9(3):e00520-18. doi: 10.1128/mBio.00520-18 (PMC5989066; doi:10.1128/mBio.00520-18)
Supplement: FIG S3 [file mbo003183910sf3.pdf]

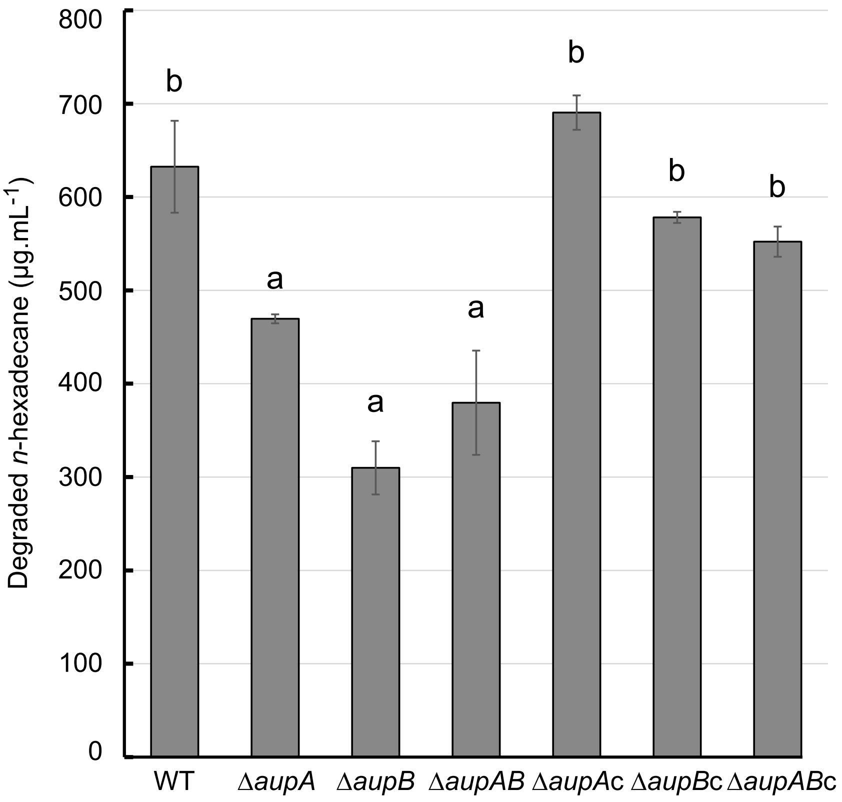

Figure S3: Hexadecane degraded in 20h in biofilm cultures of *M. hydrocarbonoclasticus* wild-type and mutant strains. WT (wild type JM1), Δ*aupA* (JM6), Δ*aupB* (JM3), Δ*aupAB* (JM4), Δ*aupA*c (JM7), Δ*aupB*c (JM8), Δ*aupAB*c (JM9). Error bars represent the standard error from three replicates. (a) Values obtained with the mutants (Δ*aupA*, Δ*aupB* and Δ*aupAB*) were statistically different with respect to the wild-type (p-value ≤ 0.03). (b) Values of the complemented strains (Δ*aupA*c, Δ*aupB*c and Δ*aupAB*c) were statistically significant different from their respective mutants (p-value$\leq$0.04) and not statistically significant different from the wild-type JM1 (p-value ≥ 0.2).
