# Supplementary material for: AupA and AupB Are Outer and Inner Membrane Proteins Involved in Alkane Uptake in Marinobacter hydrocarbonoclasticus SP17
Source: mBio. 2018 Jun 5;9(3):e00520-18. doi: 10.1128/mBio.00520-18 (PMC5989066; doi:10.1128/mBio.00520-18)
Supplement: FIG S4 [file mbo003183910sf4.pdf]

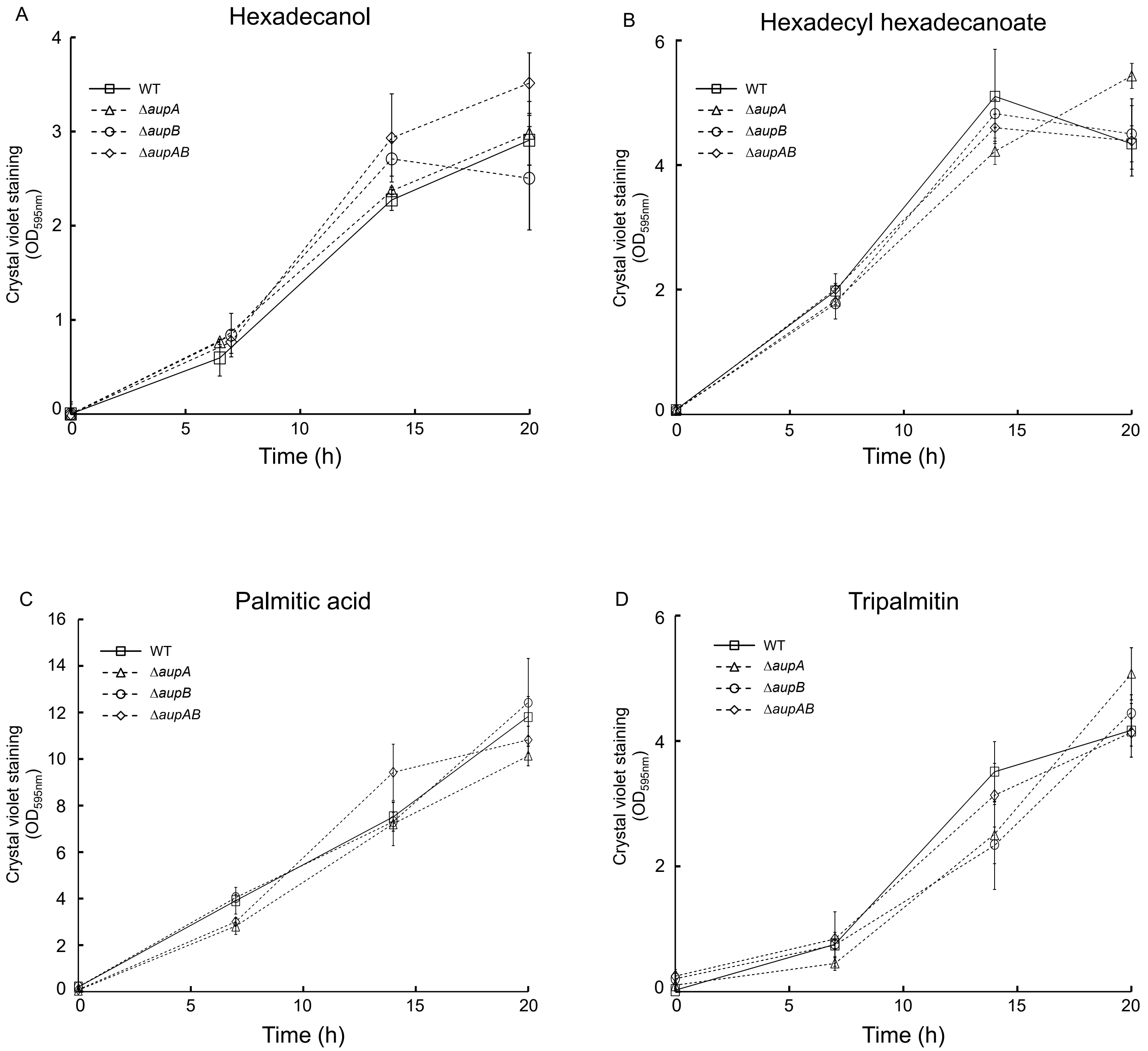

Figure S4: Kinetics of biofilm formation on hydrophobic compounds.
Biofilms were quantified by crystal violet staining. Error bars represent the standard error from three biological replicates
