# Supplementary material for: AupA and AupB Are Outer and Inner Membrane Proteins Involved in Alkane Uptake in Marinobacter hydrocarbonoclasticus SP17
Source: mBio. 2018 Jun 5;9(3):e00520-18. doi: 10.1128/mBio.00520-18 (PMC5989066; doi:10.1128/mBio.00520-18)
Supplement: FIG S5 [file mbo003183910sf5.pdf]

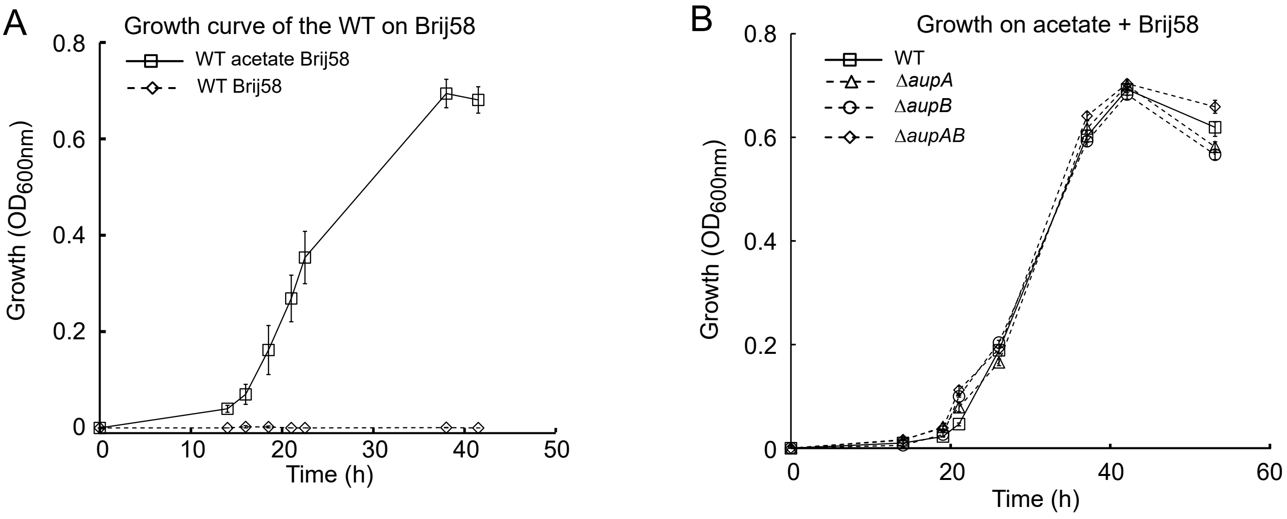

Figure S5: Kinetics of planktonic growth of *M. hydrocarbonoclasticus* strains on acetate in the presence of Brij® 58.
A) Kinetics of planktonic growth of the wild-type JM1 strain on SSW 20 mM acetate or on SSW 20 mM acetate in the presence of 4 mM Brij® 58. **B)** Kinetics of JM1 and mutant strains (Δ*aupA*, Δ*aupB* and Δ*aupAB*) on SSW 20 mM acetate in the presence of 4 mM of Brij® 58. Measurements were done on biological triplicates.
