# Supplementary material for: AupA and AupB Are Outer and Inner Membrane Proteins Involved in Alkane Uptake in Marinobacter hydrocarbonoclasticus SP17
Source: mBio. 2018 Jun 5;9(3):e00520-18. doi: 10.1128/mBio.00520-18 (PMC5989066; doi:10.1128/mBio.00520-18)
Supplement: TABLE S2 [file mbo003183910st2.pdf]

Table S2: Phylogenetic distribution of *aupA* and *aupB* genes and co-occurrence of alkane hydroxylase genes.
Genomes coding for AupA or AupB homologs were searched in the set of complete genomes of isolated strains of the IMG database using BLASTP. Criteria for positive hits were more than 30% identity and more than 80% of the query sequence aligned. * indicates possible sequencing errors or mutations resulting in fragmented proteins. Uncl unclassified; Unpub unpublished data; na not available; *alkB*: alkane 1-monooxygenase gene; *ahpG*: cytochrome P450 hydroxylase gene

	Genome Name	Tandem *aupAB*	Orphan *aupA*	Orphan *aupB*	*alkB*	*ahpG*	Hydrocarbons utilization	Habitat	Ref
Oceanospirillales	*Alcanivorax bacterium* JGI 01_B19	1			0	0		Seawater	Unpub.
	Alcanivorax bacterium JGI 01_E21	2			3	0		Seawater	Unpub.
	Alcanivorax bacterium JGI 01_H12	3			0	1		Seawater	Unpub.
	Alcanivorax borkumensis SK2	1		1	2	3	yes	Oil-contaminated seawater	1
	Alcanivorax dieselolei B5	5	1		3	3	yes	Oil-contaminated seawater	2
	Alcanivorax dieselolei KS-293	5		1	3	2		Seawater	3
	Alcanivorax hongdengensis A-11-3	3		2	4	3	yes	Oil-contaminated seawater	4
	Alcanivorax jadensis T9	3			4	4		Intertidal sediment	5
	Alcanivorax nanhaiticus 19-m-6	4			4	3		Deep sea sediment	6
	Alcanivorax pacificus W11-5	3		2	4	3	yes	Deep-sea sediment	7
	Alcanivorax sp. 43B_GOM-46m	6	2		3	2		Marine oil spill	Unpub.
	Alcanivorax sp. 97CO-5	1		1	3	2	yes	Marine sediment	8
	Alcanivorax sp. DG881	6			2	3	yes	Algal culture	Unpub.
	Alcanivorax sp. DSM 26293	4			4	3		na	Unpub.
	Alcanivorax sp. DSM 26295	5		1	4	3		na	Unpub.
	Alcanivorax sp. HI0003	4			4	1		Seawater	Unpub.
	Alcanivorax sp. HI0007	3			4	1		Seawater	Unpub.
	Alcanivorax sp. HI0011	4			4	1		Seawater	Unpub.
	Alcanivorax sp. HI0013	4			4	2		Seawater	Unpub.
	Alcanivorax sp. HI0033	4			4	1		Seawater	Unpub.
	Alcanivorax sp. HI0035	4			6	1		Seawater	Unpub.
	Alcanivorax sp. HI0044	4			3	1		Seawater	Unpub.
	Alcanivorax sp. HI0083	4			3	1		Seawater	Unpub.
	Alcanivorax sp. JRC	0		1	1 *	2 *		na	Unpub.
	Alcanivorax sp. KX64203	4			3	1		Deep-sea sediment	9
	Alcanivorax sp. NBRC 102024	4			4	3		na	Unpub.
	Alcanivorax sp. NBRC 102028	1		1	2	3		na	Unpub.
	Alcanivorax sp. P2S70	3			2	2	yes	Marine oil spill	10
	Alcanivorax sp. PN-3	3	1	2	3	1	yes	Marine oil spill	10
	Alcanivorax sp. rast	1		1	2	4		na	Unpub.
	Alcanivorax sp. sk2-jrc	1			2	3	yes	na	Unpub.
	Alcanivorax xenomutans JC109	3			3	0	yes	Shrimp pond sediment	11
	Bermanella marisrubri RED65	1			1	0		Seawater	12
	Oleibacter marinus DSM 24913	1			2	1		Seawater	13
	Oleibacter sp. HI0075	1			2	0		Seawater	Unpub.

	Genome Name	Tandem *aupAB*	Orphan *aupA*	Orphan *aupB*	*alkB*	*ahpG*	Hydrocarbon utilization	Habitat	Ref
**Alteromonadales**	*Marinobacter adhaerens* HP15	2			2	3		Marine aggregates	14
	Marinobacter algicola DG893	1			1	0	yes	Algal culture	15
	Marinobacter aquaeolei VT8	1			3	2	yes	Oil-contaminated seawater	16
	Marinobacter daepoensis DSM 16072	1			1	1		Seawater	17
	Marinobacter excellens HL-55	2			1	1		Hypersaline lake	18
	Marinobacter excellens LAMA 842	2			3	3		Deep seawater	Unpub.
	Marinobacter gudaonensis CGMCC 1.6294	1			2	0		Oil-contaminated saline soil	19
	Marinobacter hydrocarbonoclasticus ATCC 49840	1			2	2	yes	Oil-contaminated seawater	20
	Marinobacter lipolyticus SM19	2		1	2	1		Saline soil	21
	Marinobacter manganoxydans MnI7-9	2			2	1		Deep-sea hydrothermal vent	22
	Marinobacter mobilis CGMCC 1.7059	1		3	3	2		Marine sediment	23
	Marinobacter nanhaiticus D15-8W	1			1	0	yes	Marine sediment	24
	Marinobacter nitratireducens AK21	1			3	0		Seawater	25
	Marinobacter pelagius CGMCC 1.6775	1			2	1		Seawater	26
	Marinobacter salarius R9SW1	2 *			3	1		Radioactive seawater	27
	Marinobacter salsuginis SD-14B	1			3	1		Seawater	28
	Marinobacter santoriniensis NKSG1	1			2	1		Hydrothermal sediment	29
	Marinobacter similis A3d10	1 *			1	0		Seawater	27
	Marinobacter sp. CP1	1			3	0		Biocathode Biofilm	30
	Marinobacter sp. DS40M8	1 *			0	0		Seawater	31
	Marinobacter sp. DSM 26291	1			2	1		na	Unpub.
	Marinobacter sp. DSM 26671	2			3	1		na	Unpub.
	Marinobacter sp. EN3	2			3	2	yes	Marine oil spill	10
	Marinobacter sp. ES.042	1			2	0		na	Unpub.
	Marinobacter sp. ES.048	1			2	0		na	Unpub.
	Marinobacter sp. HL-58 GFM	1			1	0		Hypersaline lake	Unpub.
	Marinobacter sp. LQ44	2			2	2		Deep-sea hydrothermal vent	Unpub.
	Marinobacter sp. LV10R510-8	2			1	1		na	Unpub.
	Marinobacter sp. MCTG268	1			1	0		Marine diatom	32
	Marinobacter sp. REDSEA-S15_B16	1 *			1	0		Seawater	Unpub.
	Marinobacter sp. REDSEA-S27_B10	1 *			1	0		Seawater	Unpub.
	Marinobacter sp. YWL01	2			2	1		Aquacultural pond	Unpub.
	Marinobacter subterrani JG233	1			1	2		Mine effluent	33
	Marinobacter zhejiangensis CGMCC 1.7061	1			2	0		Marine sediment	34
	Moritella dasanensis ArB 0140	1			0	0		Glacier seawater	39
	Moritella marina ATCC 15381	1			0	0		Deep seawater	35
	Moritella sp. PE36	1			1	1		Deep seawater	Unpub.
	Moritella viscosa 06/09/139	1			0	0		Salmon	36
	Perlucidibaca piscinae DSM 21586	1			1	4	yes	Freshwater	37
**Uncl.**	*gamma proteobacterium sp.* HdN1	1			1	6	yes	Activated sludge	38
	Gammaproteobacteria TBE6_bin-46	1			2	3		na	Unpub.

References

1. Yakimov MM, Golyshin PN, Moore ERB, Abraham WR, Lünsdorf H, Timmis KN, Lang S. 1998. *Alcanivorax borkumensis* gen. nov., sp. nov., a new, hydrocarbon- degrading and surfactant-producing marine bacterium. Int J Syst Bacteriol 48:339–348.

2. Lai Q, Li W, Shao Z. 2012. Complete genome sequence of *Alcanivorax dieselolei* type Strain B5. J Bacteriol 194:6674–6674.
3. Barbato M, Mapelli F, Chouaia B, Crotti E, Daffonchio D, Borin S. 2015. Draft genome sequence of the hydrocarbon-degrading bacterium *Alcanivorax dieselolei* KS-293 isolated from surface seawater in the eastern Mediterranean sea. Genome Announc 3:e01424-15
4. Wu Y, Lai Q, Zhou Z, Qiao N, Liu C, Shao Z. 2009. *Alcanivorax hongdengensis* sp. nov., an alkane-degrading bacterium isolated from surface seawater of the straits of Malacca and Singapore, producing a lipopeptide as its biosurfactant. Int J Syst Evol Microbiol 59:1474–1479.
5. Bruns A, Berthe-Corti L. 1999. *Fundibacter jadensis* gen. nov., sp. nov., a new slightly halophilic bacterium, isolated from intertidal sediment. Int J Syst Evol Microbiol 49:441–448.
6. Lai Q, Zhou Z, Li G, Li G, Shao Z. 2016. *Alcanivorax nanhaiticus* sp. nov., isolated from deep sea sediment. Int J Syst Evol Microbiol 66:3651–3655.
7. Lai Q, Wang L, Liu Y, Fu Y, Zhong H, Wang B, Chen L, Wang J, Sun F, Shao Z. 2011. *Alcanivorax pacificus* sp. nov., isolated from a deep-sea pyrene-degrading consortium. International Journal of Systematic and Evolutionary Microbiology 61:1370–1374.
8. Luan X, Cui Z, Gao W, Li Q, Yin X, Zheng L. 2014. Genome sequence of the petroleum hydrocarbon-degrading bacterium *Alcanivorax sp*. Strain 97CO-5. Genome Announc 2:e01277-14.
9. Zhang H, Liu R, Wang M, Wang H, Gao Q, Hou Z, Gao D, Wang L. 2016. Draft genome sequence of *Alcanivorax sp.* strain KX64203 isolated from deep-sea sediments of Iheya North, Okinawa Trough. Genome Announc 4:e00872-16.
10. Overholt WA, Green SJ, Marks KP, Venkatraman R, Prakash O, Kostka JE. 2013. Draft genome sequences for oil-degrading bacterial strains from beach sands impacted by the Deepwater Horizon oil spill. Genome Announc 1:e01015-13.
11. Rahul K, Sasikala C, Tushar L, Debadrita R, Ramana CV. 2014. *Alcanivorax xenomutans* sp. nov., a hydrocarbonoclastic bacterium isolated from a shrimp cultivation pond. International Journal of Systematic and Evolutionary Microbiology 64:3553–3558.
12. Pinhassi J, Pujalte MJ, Pascual J, González JM, Lekunberri I, Pedrós-Alió C, Arahal DR. 2009. *Bermanella marisrubri* gen. nov., sp. nov., a genome-sequenced gammaproteobacterium from the Red Sea. International Journal of Systematic and Evolutionary Microbiology 59:373–377.
13. Teramoto M, Ohuchi M, Hatmanti A, Darmayati Y, Widyastuti Y, Harayama S, Fukunaga Y. 2011. *Oleibacter marinus* gen. nov., sp. nov., a bacterium that degrades petroleum aliphatic hydrocarbons in a tropical marine environment. International Journal of Systematic and Evolutionary Microbiology 61:375–380.
14. Gärdes A, Kaeppel E, Shehzad A, Seebah S, Teeling H, Yarza P, Glöckner FO, Grossart H-P, Ullrich MS. 2010. Complete genome sequence of *Marinobacter adhaerens* type strain (HP15), a diatom-interacting marine microorganism. Stand Genomic Sci 3:97–107.
15. Green DH, Bowman JP, Smith EA, Gutierrez T, Bolch CJS. 2006. *Marinobacter algicola* sp. nov., isolated from laboratory cultures of paralytic shellfish toxin-producing dinoflagellates. Int J Syst Evol Microbiol 56:523–527.
16. Huu NB, Denner EB, Ha DT, Wanner G, Stan-Lotter H. 1999. *Marinobacter aquaeolei* sp. nov., a halophilic bacterium isolated from a Vietnamese oil-producing well. Int J Syst Bacteriol 49:367–75.
17. Yoon JH, kim IG, Oh TK, Yeo SH. 2004. *Marinobacter flavimaris* sp. nov. and *Marinobacter daepoensis* sp. nov., slightly halophilic organisms isolated from sea water of the Yellow Sea in Korea. Int J Syst Evol Microbiol 54:1799–1803.
18. Nelson WC, Maezato Y, Wu Y-W, Romine MF, Lindemann SR. 2016. Identification and resolution of microdiversity through metagenomic sequencing of parallel consortia. Appl Environ Microbiol 82:255–267.
19. Gu J, Cai H, Yu S-L, Qu R, Yin B, Guo Y-F, Zhao J-Y, Wu X-L. 2007. *Marinobacter gudaonensis* sp. nov., isolated from an oil-polluted saline soil in a Chinese oilfield. International Journal of Systematic and Evolutionary Microbiology 57:250–254.
20. Gauthier MJ, Lafay B, Christen R, Fernandez L, Acquaviva M, Bonin P, Bertrand JC. 1992. *Marinobacter hydrocarbonoclasticus* gen. nov., sp. nov., a new, extremely halotolerant, hydrocarbon-degrading marine bacterium. Int J Syst Bacteriol 42:568–76.
21. Martín S, Márquez MC, Sánchez-Porro C, Mellado E, Arahal DR, Ventosa A. 2003. *Marinobacter lipolyticus* sp. nov., a novel moderate halophile with lipolytic activity. International Journal of Systematic and Evolutionary Microbiology 53:1383–1387.

22. Wang H, Li H, Shao Z, Liao S, Johnstone L, Rensing C, Wang G. 2012. Genome sequence of deep-sea manganese-oxidizing bacterium *Marinobacter manganoxydans* MnI7-9. J Bacteriol 194:899–900.
23. Huo Y-Y, Wang C-S, Yang J-Y, Wu M, Xu X-W. 2008. *Marinobacter mobilis* sp. nov. and *Marinobacter zhejiangensis* sp. nov., halophilic bacteria isolated from the East China Sea. International Journal of Systematic and Evolutionary Microbiology 58:2885–2889.
24. Gao W, Cui Z, Li Q, Xu G, Jia X, Zheng L. 2013. *Marinobacter nanhaiticus* sp. nov., polycyclic aromatic hydrocarbon-degrading bacterium isolated from the sediment of the South China Sea. Antonie van Leeuwenhoek 103:485–491.
25. Vaidya B, Kumar R, Korpole S, Tanuku NRS, Pinnaka AK. 2015. *Marinobacter nitratireducens* sp. nov., a halophilic and lipolytic bacterium isolated from coastal surface sea water. International Journal of Systematic and Evolutionary Microbiology 65:2056–2063.
26. Xu X-W, Wu Y-H, Wang C-S, Yang J-Y, Oren A, Wu M. 2008. *Marinobacter pelagius* sp. nov., a moderately halophilic bacterium. International Journal of Systematic and Evolutionary Microbiology 58:637–640.
27. Ng HJ, López-Pérez M, Webb HK, Gomez D, Sawabe T, Ryan J, Vyssotski M, Bizet C, Malherbe F, Mikhailov VV, Crawford RJ, Ivanova EP. 2014. Marinobacter salarius sp. nov. and Marinobacter similis sp. nov., Isolated from Sea Water. PLOS ONE 9:e106514.
28. Antunes A, França L, Rainey FA, Huber R, Nobre MF, Edwards KJ, da Costa MS. 2007. *Marinobacter salsuginis* sp. nov., isolated from the brine–seawater interface of the Shaban Deep, Red Sea. International Journal of Systematic and Evolutionary Microbiology 57:1035–1040.
29. Handley KM, Héry M, Lloyd JR. 2009. *Marinobacter santoriniensis* sp. nov., an arsenate-respiring and arsenite-oxidizing bacterium isolated from hydrothermal sediment. International Journal of Systematic and Evolutionary Microbiology 59:886–892.
30. Wang Z, Eddie BJ, Malanoski AP, Hervey WJ, Lin B, Strycharz-Glaven SM. 2015. Complete genome sequence of *Marinobacter sp.* CP1, isolated from a self-regenerating biocathode biofilm. Genome Announc 3:e01103-15.
31. Martinez JS, Zhang GP, Holt PD, Jung H-T, Carrano CJ, Haygood MG, Butler A. 2000. Self-assembling amphiphilic siderophores from marine bacteria. Science 287:1245–1247.
32. Gutierrez T, Whitman WB, Huntemann M, Copeland A, Chen A, Kyrpides N, Markowitz V, Pillay M, Ivanova N, Mikhailova N, Ovchinnikova G, Andersen E, Pati A, Stamatis D, Reddy TBK, Ngan CY, Chovatia M, Daum C, Shapiro N, Cantor MN, Woyke T. 2016. Genome sequence of *Marinobacter sp*. Strain MCTG268 isolated from the cosmopolitan marine diatom *Skeletonema costatum*. Genome Announc 4:e00937-16.
33. Bonis BM, Gralnick JA. 2015. *Marinobacter subterrani*, a genetically tractable neutrophilic Fe(II)-oxidizing strain isolated from the Soudan iron mine. Front Microbiol 6.
34. Huo Y-Y, Wang C-S, Yang J-Y, Wu M, Xu X-W. 2008. *Marinobacter mobilis* sp. nov. and *Marinobacter zhejiangensis* sp. nov., halophilic bacteria isolated from the East China Sea. International Journal of Systematic and Evolutionary Microbiology 58:2885–2889.
35. Kim HJ, Park S, Lee JM, Park S, Jung W, Kang J-S, Joo HM, Seo K-W, Kang S-H. 2008. *Moritella dasanensis* sp. nov., a psychrophilic bacterium isolated from the Arctic ocean. International Journal of Systematic and Evolutionary Microbiology 58:817–820.
36. Hjerde E, Karlsen C, Sørum H, Parkhill J, Willassen NP, Thomson NR. 2015. Co-cultivation and transcriptome sequencing of two co-existing fish pathogens *Moritella viscosa* and *Aliivibrio wodanis*. BMC Genomics 16:447.
37. Song J, Choo Y-J, Cho J-C. 2008. *Perlucidibaca piscinae* gen. nov., sp. nov., a freshwater bacterium belonging to the family Moraxellaceae. International Journal of Systematic and Evolutionary Microbiology 58:97–102.
38. Ehrenreich P, Behrends A, Harder J, Widdel F. 2000. Anaerobic oxidation of alkanes by newly isolated denitrifying bacteria. Arch Microbiol 173:58–64.
39. Lee SG, Koh HY, Lee JH, Kang S-H, Kim HJ. 2012. Draft genome sequence of *Moritella dasanensis* Strain ArB 0140, a psychrophilic bacterium isolated from the Arctic ocean. J Bacteriol 194:5452–5453.
